# Single‐Cell RNA Sequencing Reveals Heterogeneity of Myf5‐Derived Cells and Altered Myogenic Fate in the Absence of SRSF2

**DOI:** 10.1002/advs.202105775

**Published:** 2022-04-23

**Authors:** Ruochen Guo, Xue You, Kai Meng, Rula Sha, Zhenzhen Wang, Ningyang Yuan, Qian Peng, Zhigang Li, Zhiqin Xie, Ruijiao Chen, Ying Feng

**Affiliations:** ^1^ CAS Key Laboratory of Nutrition Metabolism and Food Safety Shanghai Institute of Nutrition and Health University of Chinese Academy of Sciences Chinese Academy of Sciences Shanghai 200031 P. R. China; ^2^ Collaborative Innovation Center for Birth Defect Research and Transformation of Shandong Province Jining Medical University Jining 272067 P. R. China

**Keywords:** apoptosis, exhaustion of myogenic pool, lineage tracing, Myf5‐derived cells, precocious differentiation, single‐cell RNA sequencing, SRSF2

## Abstract

Splicing factor SRSF2 acts as a critical regulator for cell survival, however, it remains unknown whether SRSF2 is involved in myoblast proliferation and myogenesis. Here, knockdown of SRSF2 in myoblasts causes high rates of apoptosis and defective differentiation. Combined conditional knockout and lineage tracing approaches show that Myf5‐cre mice lacking SRSF2 die immediately at birth and exhibit a complete absence of mature myofibers. Mutant Myf5‐derived cells (tdtomato‐positive cells) are randomly scattered in the myogenic and non‐myogenic regions, indicating loss of the community effect required for skeletal muscle differentiation. Single‐cell RNA‐sequencing reveals high heterogeneity of myf5‐derived cells and non‐myogenic cells are significantly increased at the expense of skeletal muscle cells in the absence of SRSF2, reflecting altered cell fate. SRSF2 is demonstrated to regulate the entry of Myf5 cells into the myogenic program and ensures their survival by preventing precocious differentiation and apoptosis. In summary, SRSF2 functions as an essential regulator for Myf5‐derived cells to respond correctly to positional cues and to adopt their myogenic fate.

## Graphical abstract

Splicing factor SRSF2 plays a key role in myoblast proliferation and differentiation. Loss of SRSF2 in Myf5‐derived cells causes severe myogenic defects. Lineage tracing and single‐cell RNA sequencing shows that Myf5‐derived cells have great heterogeneity. SRSF2 regulates Myf5 cells to enter the myogenic program and ensures their survival by preventing immature differentiation and apoptosis.

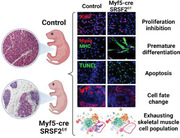

## 1 Introduction

In the course of skeletal muscle development, the lips of the dermomyotome can mature into the myotome that is a segmented structure called somites. They contain muscle progenitor cells (MPCs) highly expressing the paired box transcription factors Pax3 and Pax7.^[^ ^1^ ^]^ It is generally believed that MPCs establish their myogenic fate by forming committed myoblasts with expression of one or a few myogenic regulatory factors (MRFs).^[^ ^2^ ^]^ Following myogenesis involves withdrawal of myoblasts from the cell cycle and differentiation into mononucleated myocytes, and later fusion to form multinucleated myofibers. MRFs compose of Myf5, MyoD, Myog and Myf6, which function downstream of Pax3 and Pax7. Gene targeting studies revealed that MyoD and Myf5 are both considered to be myogenic determination genes,^[^ ^3^ ^]^ whereas Myog and Myf6 are regarded as differentiation genes that play a critical role in differentiating myocytes and myofibers.^[^ ^4^ ^]^

Myf5 transcripts are first detected on the embryonic day E8.0 in the dorsal cells of the somites, which occurs about 2 days earlier than MyoD expression.^[^ ^5^ ^]^ During this early stage, MPCs that have activated expression of Myf5 still remain multipotent, as loss of Myf5 induces aberrant migration and mislocalization of these cells, resulting in altered cell fate.^[^ ^5b^ ^]^ In the absence of Pax3 and Myf5, MyoD is not activated and skeletal muscles are ablated in the body.^[^ ^6^ ^]^ All these findings strongly indicate that Myf5 acts upstream of MyoD to direct embryonic multipotent cells into the myogenic lineage. On the other hand, Myf5^+^ cells have been reported to contribute to fat formation^[^ ^7^ ^]^ and a portion of the ribs and cervical vertebrae.^[^ ^8^ ^]^ Moreover, previous research has indicated that these Myf5‐derived cells can generate a functional neural crest cell type,^[^ ^9^ ^]^ despite it is still under controversy.^[^ ^10^ ^]^

The activity MRFs is tightly coupled to cell cycle control, and abnormal regulation of cell cycle can induce an uncontrolled myogenic differentiation. The retinoblastoma tumor suppressor protein (Rb) plays a key role in controlling cell cycle progression, and Rb‐deficient mice exhibit high rates of apoptosis and an almost complete absence of myofibers.^[^ ^11^ ^]^ Induction of cyclin‐dependent kinase (Cdk) inhibitors such as p21 and p57 is required for terminal cell cycle withdrawal during skeletal muscle differentiation. Mice lacking both p21 and p57 fail to form myotubes, display increased proliferation and apoptotic rates of myoblasts.^[^ ^12^ ^]^

Genetic lineage tracing is a useful tool for labeling endogenous cells and tracking cell fate during embryonic development.^[^ ^13^ ^]^ In this study, we introduced a Cre‐dependent tdtomato reporter gene tracking system, in which Rosa26‐tdTomato (tdT) mice were used to cross with Myf5‐Cre mice.^[^ ^14^ ^]^ Combined with single cell RNA sequencing (scRNA‐seq) technology, we identified and characterized the transcriptome characteristics of Myf5‐derived cells during embryonic myogenesis. Surprisingly, we observed that Myf5‐derived cells showed a wide range of heterogeneity. While long‐term expression of Myf5 was only detected in the skeletal muscle cells, transient activity of Myf5 contributed to various non‐myogenic cell lineages including mesenchymal cells, chondrocytes, neural cells, endothelial cells, osteoblasts, Schwann cells, and immune cells.

SRSF2 belongs to the family of SR proteins and acts as a crucial regulator of constitutive and alternative pre‐mRNA splicing. Our previous work has demonstrated that absence of SRSF2 resulted in large number of hepatocyte apoptosis and acute liver failure.^[^ ^15^ ^]^ Knockdown of SRSF2 caused severe death of live cancer cells.^[^ ^16^ ^]^ These findings strongly emphasize the critical role of SRSF2 in cell survival. In addition, SRSF2 is also reported to interact with E2F1 and stimulates its transcriptional control of cell cycle target genes.^[^ ^17^ ^]^

In this study, we find that Myf5‐cre mice lacking SRSF2 die at birth, display high rates of apoptosis of myoblasts and total loss of mature myofibers. Significantly, we observe a scattered and disordered arrangement of SRSF2‐depleted tdT cells in the limbs and intercostal muscles, indicating loss of the community effect for skeletal muscle differentiation.^[^ ^18^ ^]^ scRNA‐seq further reveals that non‐myogenic cells are increased at the expense of skeletal muscle cells in the absence of SRSF2. Moreover, differential gene expression analysis shows that differentiation markers Myog and p21 were upregulated, accompanied by abnormal expression of Pax7 and Myf5 during the differentiation pseudotime of mutant versus control. Finally, we demonstrate that knockdown of SRSF2 causes precocious differentiation of myoblasts and leads to exhaustion of the progenitor pool. Taken together, SRSF2 is necessary for Myf5 cells to adopt their myogenic fate during embryonic myogenesis.

## 2 Results

### 2.1 SRSF2 is Critical for Proliferation and Differentiation of Myoblasts In Vitro

**Figure 1:**
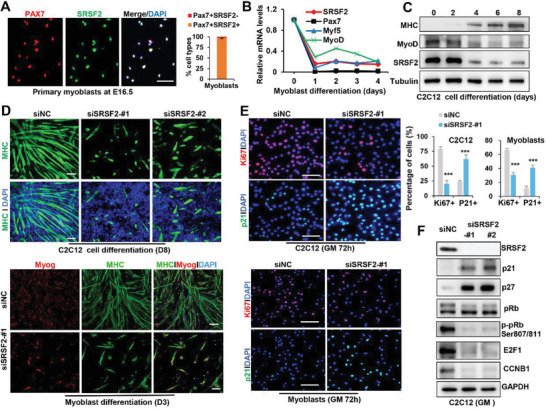
SRSF2 knockdown caused severe proliferation and differentiation defects of both myoblasts and C2C12 cells. A) Representative confocal images of Pax7 (red), SRSF2 (green), and DAPI (blue) immunostaining in primary myoblasts purified from WT embryos at E16.5. Scale bars, 50 µm. 2 × 10^3^ cells were seeded on the coverslips and stained with indicated antibodies. Qualification of Pax7 and SRSF2 colocalization in myoblasts was shown on the right (*n* = 3). Data were presented as the mean ± SD. B) Relative mRNA levels of indicated genes during primary myoblast differentiation. The results were plotted as the mean ± SD (*n* = 3). C) Representative WB analysis of MHC, MyoD, and SRSF2 during differentiation of C2C12 cells (*n* = 3). Cells were induced into differentiation and whole cell lysates were isolated at indicated time and subjected to WB analysis. D) Representative confocal images of MHC (green), Myog (red), and DAPI (blue) immunostaining in C2C12 cells and primary myoblasts (*n* = 3). Scale bars, 100 µm. Cells were transfected with indicated siRNAs, then induced into differentiation for 8 days (C2C12 cells) or 3 days (myoblasts), respectively. E) Representative confocal images of Ki67 (red) and p21 (green) immunostaining in C2C12 cells and primary myoblasts. Scale bars, 100 µm. After transient transfection with indicated siRNAs for 48 h, cells were reseeded on the coverslips at density of 1 × 10^4^ cells for siNC group and 2 × 10^4^ cells for siSRSF2‐#1 group, respectively, followed by immunostaining. Qualification of Ki67^+^ and p21^+^ cells was shown on the right (*n* = 3). Data were presented as the mean ± SD. 2‐tailed Student's *t*‐test. *** indicates *p* < 0.001. F) Representative WB analysis of C2C12 cells after RNA interference as indicated for 48 h (*n* = 3).

To explore the role of SRSF2 in myogenic differentiation, we isolated primary myoblasts from wild‐type mice at E16.5 and performed immunostaining for Pax7, Myf5, MyoD and SRSF2. SRSF2 was expressed in the majority of Pax7‐positive or Myf5‐positive myoblasts, as well as in ≈80% MyoD‐positive cells (**Figure** **1**A; Figure S1A,B, Supporting information). Then we induced myoblasts into differentiation in vitro, and performed RT‐qPCR analysis. mRNA levels of SRSF2 declined sharply after differentiation, which was highly similar to the expression pattern of Myf5 and MyoD (Figure 1B). Protein levels of SRSF2 was similarly decreased in immortalized C2C12 cells upon differentiation, and the differentiation marker Myog‐expressing cells also showed weak signals for SRSF2 (Figure 1C; Figure S1C, Supporting Information).

Next we transfected C2C12 cells and myoblasts with control siRNA (siNC) or SRSF2‐specific siRNAs (siSRSF2‐1#, ‐2#) for 48 h, then switched cells from growth medium (GM) to differentiation medium (DM). Immunostaining with Myog and/or myosin heavy chain (MHC) revealed that knockdown of SRSF2 dramatically impaired myogenic differentiation of C2C12 cells and myoblasts (Figure 1D). Long multinucleated myotubes typically seen in control cells were absent, instead only a few short, unaligned myotubes were observed following SRSF2 knockdown. In addition, siSRSF2 significantly decreased intensity of the proliferation marker Ki67 and increased p21 signals, compared to control siRNA (Figure 1E). Western blot (WB) analysis further demonstrated that SRSF2 knockdown led to activation of MyoD‐dependent genes such that levels of p21, p57, and upregulation of hypophosphorylated pRb. On the other hand, E2F1 itself and E2F1‐dependent cell cycle genes such as Ccnb1 were switched off, as well as deceased levels of phosphorylated pRb (p‐pRb) (Figure 1F). In addition, the effects of SRSF2 overexpression were relatively modest on cell proliferation, probably because of SRSF2's relatively high abundance in cells (Figure S2, Supporting Information). Taken together, these data provided evidence that SRSF2 plays a key role in regulating proliferation and differentiation of myoblasts in vitro.

### 2.2 Loss of SRSF2 Caused Severe Skeletal Muscle Defects

**Figure 2:**
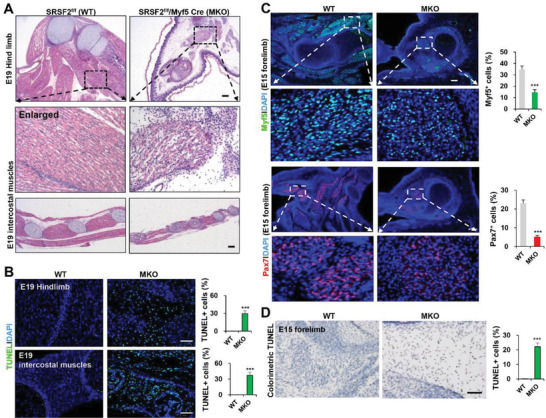
SRSF2 is essential for skeletal muscle development. A) Representative hematoxylin and eosin (HE) staining of hindlimbs and intercostal muscles at E19 (*n* = 5). Scale bars, 100 µm. Note that black dotted boxes are shown enlarged in the middle panels. B) Representative confocal images of fluorometric TUNEL staining with sections at E19. Scale bars, 100 µm. The percentage of TUNEL‐positive cells was shown on the right bar graphs (*n* = 3). C) Representative confocal images of Myf5 (green) and Pax7 (red) immunostaining with forelimb sections at E15. Scale bars, 100 µm. The percentage of Myf5^+^ and Pax7^+^ cells was shown on the right bar graphs (*n* = 3). D) Representative images of colorimetric TUNEL staining of sections at E15. Scale bars, 100 µm. The percentage of TUNEL‐positive cells was shown on the right bar graphs (*n* = 3). All data are shown as the mean ± SD. 2‐tailed Student's *t*‐test. *** *p* < 0.001.

To further investigate the requirement for SRSF2 in myogenesis, we inactivated *Srsf2* gene in myoblasts by crossing SRSF2^f/f^ mice (WT) with Myf5‐Cre mice to generate SRSF2^f/f^/Myf5‐Cre mice (MKO) (Figure S3A, Supporting Information). Newborn MKO pups died shortly after birth, and they displayed apparent skeletal muscle and rib defects, as well as significant weight loss (Figure S3B, Supporting Information). Protein levels of SRSF2 were significantly decreased in the hindlimb muscles of MKO mice, compared to control (Figure S3C, Supporting Information). Histological examination of skeletal muscles at the embryonic day 19 (E19) revealed the presence of severe differentiation defects (**Figure** **2**A). Hindlimb muscles exhibited a dramatic reduction in mass with a complete absence of mature fibers compared with littermate controls. Paralleled myofibers typically seen in WT controls were absent and replaced by short irregular fibers in MKO mice. Those muscle defects were also observed in the forelimb, intercostals and tongues, but the diaphragm was just thinning that seemed less affected in the MKO mice (Figure S3D, Supporting Information). Moreover, TUNEL analysis showed high levels of apoptosis in the hindlimb and intercostals of mutant mice at E19 (Figure 2B).

At E15, mutant embryos showed severe edema and a strong reduction in the body wall muscle (Figure S4, Supporting Information). Immunostaining with forelimb sections at E15 revealed that there are large amount of myogenic precursor cells/myoblasts that expressed Pax7 and Myf5 in the control muscles, which were significantly decreased in the MKO embryos (Figure 2C). Consistently, a large number of apoptotic cells were present in the mutant forelimb (Figure 2D). These results strongly indicated that mutant muscle cells were undergoing abnormal apoptosis during embryonic myogenesis.

### 2.3 Abnormal Distribution and Differentiation of Myf5‐Derived Cells in the Mutant Mice

**Figure 3:**
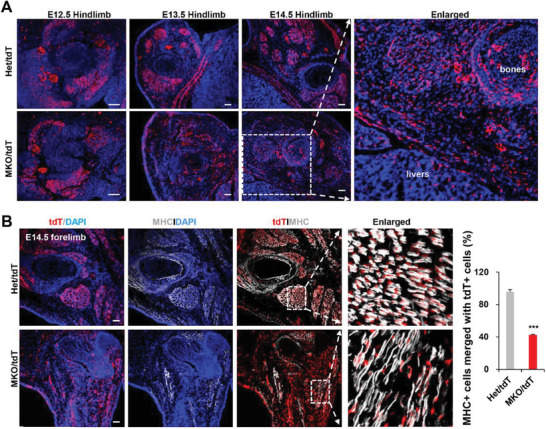
Loss of SRSF2 resulted in aberrant clustering and myogenic differentiation of Myf5‐derived cells. A) Representative confocal images of tdT (red) immunostaining with sections from Het/tdT and MKO/tdT embryos at E12.5, E13.5, and E14.5 (*n* = 3). Scale bars, 100 µm. B) Representative confocal images of tdT (red) and MHC (white) immunostaining with sections at E14.5. Scale bars, 100 µm. Note that white dotted boxes are shown enlarged in the right panels. Qualification was shown on the right bar graph (*n* = 3). Data were presented as the mean ± SD. 2‐tailed Student's *t*‐test. *** *p* < 0.001.

To order to track the myogenic fate of Myf5‐derived cells, we crossed heterozygous mice (Het:SRSF2f/W/Myf5‐cre) with a mouse line harboring a Rosa26‐lSL‐tdTomato allele to specifically label Myf5‐expressed cells by tdTomato (tdT) (Figure S5, Supporting Information). We first prepared transverse sections of limbs from control and MKO/tdT mice at E12.5, E13.5, and E14.5, respectively. tdT staining revealed that majority of tdT+ cells (Myf5‐derived cells) were located in the prospective muscle regions, arranged in homogeneous coherent groups sharply demarcated from adjacent non‐muscle cells in control mice at the three different stages (**Figure** **3**A). This is called a community effect during muscle development, which is believed to activate muscle gene expression in uniform cell populations.^[^ ^18^ ^]^ However, such effect was completely disrupted in E13.5 and E14.5 mutant mice, although relatively normal pattern was observed at E12.5 mice. Enlarged images further confirmed that tdT+ cells were wildly scattered throughout the limb region, and they even migrated into the livers and bones of mutant mice (Figure 3A).

Myf5‐derived cells in control mice displayed normal myogenic commitment and differentiation to functional myofibers, as addressed by immunostaining for MHC (Figure 3B; Figures S6A, 7A, and 8A, Supporting Information). Importantly, we also observed that they remained closely associated (merged) with MHC‐positive fibers. In contrast, majority of tdT+ cells in the MKO mice couldn't undergo normal developmental program to acquire a myogenic fate at both E13.5 and E14.5, as a few MHC‐positive fibers were observed and ≈40–50% myofibers were not merged with tdT+ cells, indicating aberrant differentiation of mutant tdT+ cells (Figure 3B; Figure S7A, Supporting Information). One day earlier at E12.5, there was not much differences of MHC staining between control and MKO mice (Figure S6A, Supporting Information), indicating that the first wave of myogenic differentiation was relatively normal in the mutant mice.

### 2.4 Progressive Loss of MPCs Were Observed in the MKO Mice

**Figure 4:**
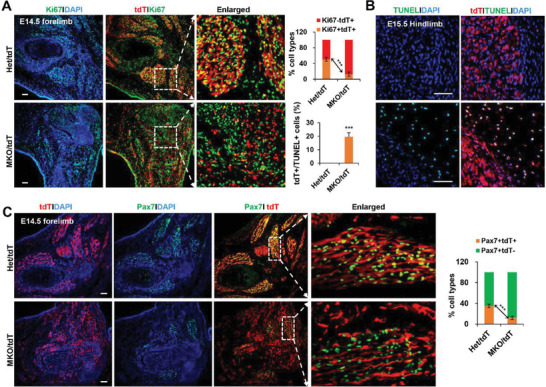
Decreased proliferation of Myf5‐derived cells and decreased expression of Pax7 in the mutant forelimbs. A) Representative confocal images of tdT (red) and Ki67 (green) immunostaining of forelimb sections at E14. Scale bars, 100 µm. White dotted boxes are shown enlarged in the right panels. Quantitative analysis of ki67^+^ and tdT^+^ cells was shown on the top bar graph (*n* = 3). B) Representative confocal images of tdT and TUNEL staining with forelimb sections at E15.5. Scale bars, 100 µm. Quantitative analysis of TUNEL and tdT double positive cells was shown on the bottom bar graph to the left of pictures (*n* = 3). C) Representative confocal images of tdT (red) and Pax7 (green) immunostaining with forelimb sections at E14.5. Scale bars, 100 µm. Note that white dotted boxes are shown enlarged in the right panels. Quantitative analysis of Pax7 and tdT cells was shown on the right bar graph (*n* = 3). All data are shown as the mean ± SD. 2‐tailed Student's *t*‐test. *** *p* < 0.001.

Next we wanted to examine the proliferation rates of tdT+ cells by immunostaining for Ki67. At E14.5, we observed up to a 5‐fold decrease in the number of Ki67/tdT double‐positive cells in control limbs versus the mutant (**Figure** **4**A; Figure S8A, Supporting Information). Meanwhile, we detected a large number of tdT+ apoptotic cells in the mutant mice, but only a few apoptotic cells were present in control mice (Figure 4B). More importantly, a massive reduction of Pax7+ cells were observed in the mutant mice at E13.5 and E14.5, which occurred concomitantly with the significantly decreased MHC‐positive fibers (Figure 4C; Figures S7B and 8B, Supporting Information). The remaining Pax7+ cells have lost their regular contact with tdT+ cells, 20% of double‐positive cells were observed in the MKO mice, compared to ≈40% in the control mice at E13.5 (Figure S7B, Supporting Information). With the increasing age, progressively fewer double‐positive cells were detected at E14.5 limbs (Figure 4C; Figure S8B, Supporting Information). Apparently, the pool of MPCs underwent an accelerated depletion, which could be due to dispersed distribution and increased apoptosis of tdT+ cells.

### 2.5 scRNA‐seq Revealed Diverse Cell Populations of tdT^+^ Cells and Identified Exhausting Skeletal Muscle Cell Population in the Mutant Mice

**Figure 5:**
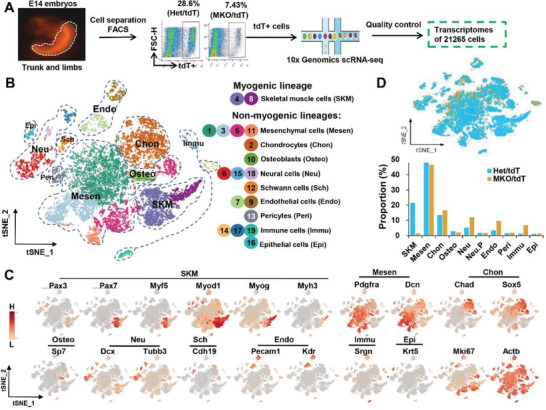
scRNA‐seq identifies distinct cell lineages of Myf5‐derived cells. A) Scheme of single cell preparation from control and MKO/tdT truncated embryos at E14, FACS isolation for tdT^+^ cell, and scRNA‐seq. B) t‐SNE plot of combined dataset with cells colored by cell types. C) Expression of representative marker genes in distinct cell clusters. D) t‐SNE plot of overlapping control and MKO/tdT datasets (top), and the proportion of different cell types in control and MKO/tdT samples (bottom).

To characterize cell fates and dynamics of Myf5‐derived cells with or without SRSF2 in skeletal muscle development, we isolated mononuclear cells from E14 Het/tdT and MKO/tdT embryos that were decapitated and eviscerated, then separated tdT^+^ cells by flow cytometry and applied them to scRNA‐seq (**Figure** **5**A). At this stage we found a significant decrease in the number of tdT^+^ cells in the mutant embryos compared to control (7.43% vs 28.6%). After quality control (Figures S9 and S10, Supporting Information), we retained a total of 21 265 transcriptomes, including 9439 control and 118 226 MKO cells for the downstream analysis (Figure 5A).

The t‐distributed stochastic neighbor embedding (t‐SNE) plots revealed 19 cell clusters, which displayed distinct transcriptional programs (Figure 5B; Figure S11A, Supporting Information). Based on established lineage‐specific marker genes (Figure 5C) and cluster‐cluster distance analysis (Figure S11B, Supporting Information), we assigned these 19 clusters into 10 different cell types (Figure 5B, right panel). Both cluster 4 (C4) and C8 expressed skeletal muscle cell (SKM) markers, such as *Pax7*, *Myf5*, *Myod1*, *Myog*, and *Myh3*, which constitute the myogenic lineage. On the other hand, the remaining 9 cell types contributed to non‐myogenic lineages. Mesenchymal cells (Mesen) composing of C1, C3, C5, and C11, were enriched for *Pdgfra* and *Dcn*; chondrocytes (Chon:C2) highly expressed *Chad5* and *Sox5*; Osteoblasts (Osteo: C10) expressed *Sp7*; neural cells (Neu: C6, C15 and C18) were enriched for *Dcx* and *Tubb3*; Schwann cells (Sch: C12) expressed *Cdh19*; endothelial cells (Endo: C7 and C9) highly expressed *Pecam1* and *Kdr*; immune cells (Immu: C14, C17, and C19) expressed *Srgn* and epithelial cells (Epi:C16) specifically expressed *Krt5*. While the house‐keeping gene *Atcb* was expressed in all the cell populations, *Mki67* was mainly enriched in SKM and Mesen groups (Figure 5C). These results revealed high heterogeneity of Myf5‐derived cells during the development. But the current expression of Myf5 was predominantly observed in the SKM population.

We next analyzed the proportions of these distinct cell types in control and mutant samples (Figure 5D). Approximately 21.44% of tdT^+^ cells became myogenic in the control, however only a small percentage of cells (≈1.43%) belong to the SKM in the mutant mice. In contrast, the proportions of Neu, Endo, and Immu cells were significantly increased in the KO mice, perhaps resulting from the excessive loss of SKM cells. Besides, the Mesen group occupied ≈49% of total Myf5‐derived cells in either control or mutant mice, but they were not totally overlapped between the two samples (Figure 5D, top panel). Taken together, loss of SRSF2 significantly changed cell proportions among Myf5‐derived cells.

### 2.6 Trajectory Analysis of SKM Populations Revealed Aberrant Proliferation and Differentiation of Myoblasts in the Absence of SRSF2

**Figure 6:**
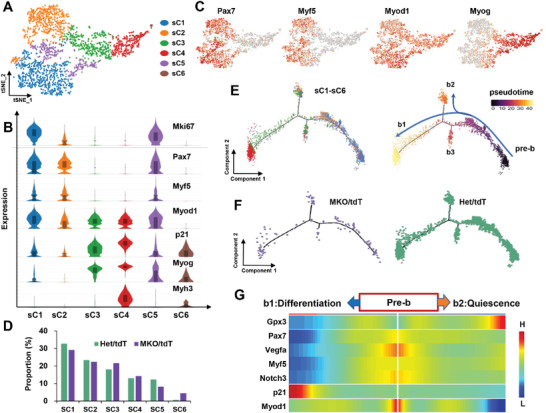
Subclustering of SKM cells and pseudotime analysis. A) t‐SNE projections of subclustered SKM cells, labeled in different colors. B) Violin plot indicating gene expression signatures in SKM cell subclusters. C) tSNE plot showing the expression of marker genes of Pax7, Myf5, Myod1, and Myog. D) the proportion of subclusters in control and MKO/tdT samples. E) Pseudotime‐ordered analysis of SKM cells, constructed by Monocle2. Right panel was colored by the subclusters. Left panel was colored by the pseudotime order. F) Pseudotime‐ordered analysis of SKM cells, from the mutant (left panel) and control (right panel) samples. G) Heatmap showing the dynamic expression of the selected genes along the trajectories b1 and b2.

We next performed unsupervised clustering on the SKM population, which identified a total of six unique subclusters that we labeled as sC1 to sC6 (**Figure** **6**A). Specifically, sC1 cells expressed high levels of *Pax7, Myf5*, and *Myod1*, also enriched for *Mki67*, thus we defined sC1 as activated myoblasts. sC2 cells were less activated than sC1 cells, as they expressed relatively low levels of these markers. sC3, sC4, and sC6 were identified as differentiated populations based on expression of differentiation and mature fiber markers such as *p21*, *Myog*, and *Myh3*. And sC5 cells, enriched for both activation and differentiation makers, might represent transitional states (Figure 6B,C). In addition, we noticed an increased proportion of differentiated cells and a decreased proportion of activated cells in the mutant compared to the control (Figure 6D).

We then used Monocle2^[^ ^19^ ^]^ to analyze dynamic cell transitions during the myogenesis, which arranged the six subclusters along one major trajectory and two smaller trajectories (Figure 6E). Majority of sC1 and sC2 plotted tightly together at the start of pseudotime (pre‐b), and only small fraction of cells ended at the trajectory b2. Differentiated cells located toward or at the end of the major branch (b1) and small branch (b3), whereas sC5 cells did not exhibit a spatial bias. Even though there were only small number of mutant cells, they were more inclined to be distributed towards b1 than the control cells (Figure 6F). We next investigated the dynamic changes of indicated gene expression along the branches b1 and b2 (Figure 6G). High expression of *Pax7*, *Myf5* and *Myod1*was observed at the start of pseudotime, whereas *p21* was restricted to the end of b1, and the quiescent marker *Gpx3* was enriched at the end of b2, together with the total loss of *Myod1*. This strongly suggested that activated myoblasts either go to differentiation, or enter into the quiescence.

**Figure 7:**
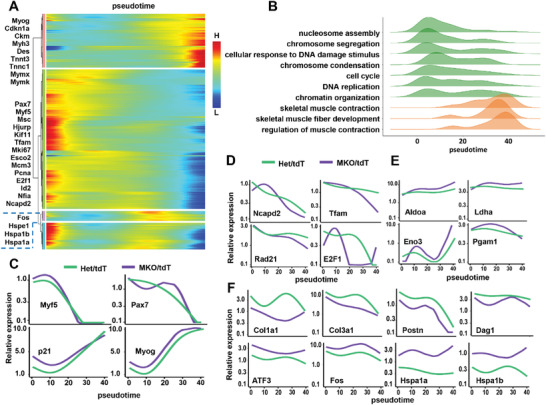
Analysis of SKM cell transition states in MKO/tdT and Het/tdT samples. A) Heatmap showing the dynamic changes in gene expression along the differentiation pseudotime. Representative genes were listed on the left. B) The enriched GO terms (biological processes) changes with the degree of enrichment along the pseudotime. The horizontal axis is the pseudo‐time and the vertical axis is an enrichment item. C) Plots showing the expression scores for indicated myogenesis markers during the pseudotime order. D) Plots showing the expression scores for genes involved in karyokinesis and proliferation during the pseudotime order. E) Plots showing the expression scores for glycolysis‐related genes in pseudotime order. F) Plots showing the expression scores for genes related with cytoskeleton and stress‐response during the pseudotime order.

Quiescent cells account for satellite cells, and they were mainly responsible for postnatal skeletal muscle development.^[^ ^20^ ^]^ We next focused our attention on the differentiation trajectory. We excluded portion of the quiescent cells and performed the dynamic changes of gene expression along the differentiation process (**Figure** **7**A; Figure S12, Supporting Information). And representative GO terms in biological process was illustrated in Figure 7B. Down‐regulated genes were enriched in MPC markers, nucleosome assembly, cell cycle, and DNA replication et al such as *Pax7, Myf5, E2f1, Kif11, Tfam, Mki6, Esco2*. Up‐regulated genes are mainly related with skeletal muscle contraction and fiber development, for example *Myog*, *Ckm*, and *Myh3*. We next analyzed the expression levels of genes along the pseudotime in the mutant and control separately. Interestingly, we observed that expression of *Myf5* was significantly upregulated at the earlier pseudotime accompanied by increased levels of *Myog* and *Cdkn1a* in the mutant, compared to the control, whereas levels of *Pax7* first decreased and then rapidly increased in the mutant (Figure 7C). Moreover, proliferation‐related genes *Tfam*, *Rad21*, *Ncapd2*, and *E2F1* were all dysregulated, while glycolysis‐related genes *Eno3*, *Pgam2*, *Aldoa*, and *Ldha* were all upregulated in the mutant (Figure 7D,E). Consistently, cytoskeleton‐related genes *Col1a1*, *Col3a1*, *Postn*, and *Dag1* were downregulated and expression of stress genes *ATF3*, *Fos*, *Hspa1a*, and *Hspa1b* were significantly increased along the pseudotime in the mutant compared with the control (Figure 7F,G). These data strongly indicated that myoblasts display abnormal proliferation and differentiation in the absence of SRSF2.

### 2.7 Precocious Differentiation and Depletion of the Progenitor Pool Were Observed in the Mutant Mice and Myoblasts

**Figure 8:**
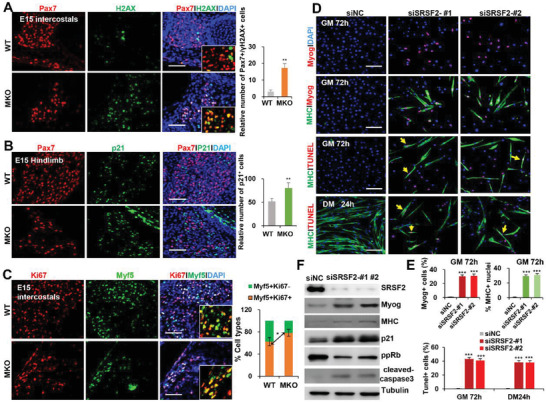
Loss of SRSF2 resulted in abnormal proliferation, exhaustion and precocious differentiation of myoblasts. A) Representative confocal images of Pax7 (red) and *γ*H2AX (green) immunostaining of intercostal sections at E15. Scale bars, 100 µm. Quantification of Pax7 and *γ*H2AX cells per area (0.1 mm^2^) are shown on the right bar graph (*n* = 3). B) Representative confocal images of Pax7 (red) and p21 (green) immunostaining of hindlimb sections at E15. Scale bars, 100 µm. Quantification of p21 cells per area (0.1 mm^2^) was shown on the right bar graph (*n* = 3). C) Representative confocal images of Myf5 (red) and Ki67 (green) immunostaining with intercostal at E15. Scale bars, 100 µm. Quantitative analysis of Myf5 and Ki67 cells was shown on the right bar graph (*n* = 3). D) Representative confocal images of Myog (red), MHC (green) and TUNEL (red) staining of primary myoblasts. Scale bars, 100 µm. After 48 h of transient transfection, primary myoblasts were kept in growth medium (GM) for another 24 h or transformed into differentiation medium (DM) for 24 h. Cells in GM were stained for Myog (red), MHC (green), or TUNEL (red), respectively, while cells in DM were stained with MHC (green) and TUNEL (red). E) Quantitative analysis of Myog (GM 72 h), MHC (GM 72 h) and TUNEL (GM 72 h and DM 24 h) was shown in (*n* = 3). F) Representative WB analysis in primary myoblasts after RNA interference as indicated for 72 h (*n* = 3). All data are shown as the mean ± SD. 2‐tailed Student's *t*‐test. *p < 0.05.**p < 0.01.***p < 0.001.

In order to validate scRNA‐seq data, we first performed immunostaining for Pax7 and the DNA‐damage marker *γ*H2AX using hindlimb and intercostal sections prepared from E15 MKO and WT embryos. Although number of Pax7^+^ cells in the mutant muscles were greatly dropped, more than 10 times Pax7^+^/*γ*H2A^+^ cells were observed in the mutant than in the control (**Figure** **8**A), suggesting that mutant Pax7^+^cells tend to die easily, compared to control cells. Moreover, levels of p21 were significantly increased in the mutant muscles (Figure 8B). And Myf5^+^ cells were undergoing expansion more extensively in the KO mice than in the control, as addressed by Myf5 and Ki67 double staining (Figure 8C). Consistently, severely impaired myogenesis occurred, as diminished expression of Myog, MHC and embryonic myosin chain (MYH3) was observed in the mutant mice at E15 (Figure S13, Supporting Information).

Next we transfected primary myoblasts with siNC or siSRSF‐#1, #2 in GM for 72 h, then stained for Myog and MHC. A lot of Myog^+^ myoblasts and MHC^+^ myotubes were observed in SRSF2‐knockdown cells, indicating that SRSF2 knockdown caused precocious myogenic differentiation (Figure 8D,E, GM 72 h). However, upon differentiation, SRSF2‐knockdown myoblasts displayed extremely decreased differentiative capacity (Figure 8D, DM 24 h). TUNEL analysis further confirmed high rates of apoptosis within differentiated myotubes (yellow arrows) as well as within undifferentiated myoblasts following SRSF2 depletion (Figure 8D,E, DM 24 h and GM 72 h). Consistent with phenotypes, WB analyis demonstrated that siSRSF2 induced the expression of Myog and MHC, upregulation of p21 and the cell‐death marker cleaved caspase 3, while decreased ppRb levels, compared to control siRNA (Figure 8F). Taken together, SRSF2 knockdown caused precocious differentiation and high rates of apoptosis in primary myoblasts.

### 2.8 SRSF2 Regulated Transcription of Cell‐Cycle/Cytoskeleton‐Related Genes and p21 Depletion Rescued Defects Seen in SRSF2 Knockdown

**Figure 9:**
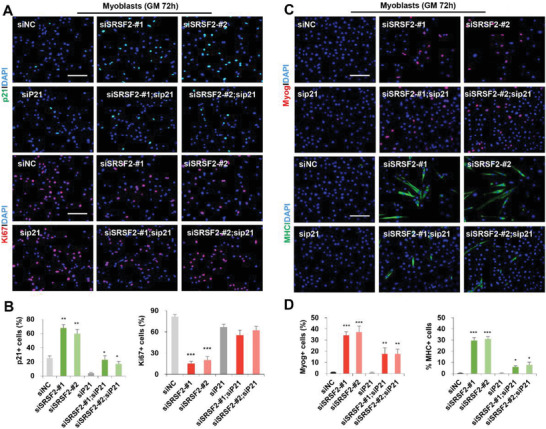
Knockdown of p21 could rescue defects of proliferation and differentiation induced by SRSF2 depletion. A) Representative confocal images of p21 and Ki67 immunostaining in primary myoblasts Myoblasts were transfected with siRNAs as indicated for 48 h, and then reseeded on coverslips (2 × 10^4^ cells for siSRSF2‐#1 and siSRSF2‐#1 groups, 1 × 10^4^ cells for other groups) before staining. Qualification of p21^+^ cells and Ki67^+^ cells are shown in (B) (*n* = 3). C) Representative confocal images of Myog, MHC immunostaining of primary myoblasts. Scale bars, 100 µm. Qualification of Myog+ cells and MHC+ cells are shown in (D) (*n* = 3). All data were shown as the mean ± SD. 2‐tailed Student's *t*‐test. *p < 0.05.**p < 0.01.***p < 0.001.

The skeletal muscle defects of SRSF2‐null mice are reminiscent of the Rb null phenotypes.^[^ ^11^ ^]^ We next wanted to examine whether SRSF2 mediated its effects on myogenesis through cell cycle‐related genes. Given that p21 was significantly upregulated in the mutant muscle and also in SRSF2‐knockdown myoblasts, we decided to test if knockdown of p21 could rescue defects seen in SRSF2 depletion. To this end, we co‐transfected primary myoblasts with siRNA against p21 (sip21) and siSRSF2, followed by immunostaining for p21, Ki67, Myog, or MHC, respectively. As shown, levels of p21 were efficiently reduced in myoblasts transfected with both siSRSF2 and sip21, compared to siSRSF2‐transfected cells. Downregulation of p21 resulted in increased Ki67 positivity in double siRNA‐transfected cells, compared to siSRSF2‐transfected cells, although it had no significant effects on proliferation of cells only transfected with sip21 (**Figure** **9**A,B). Moreover, knockdown of p21 significantly rescued precocious differentiation induced by downregulation of SRSF2, as decreased staining for Myog and MHC was observed in double knockdown cells, in comparison with SRSF2‐knockdown cells (Figure 9C,D). In summary, these results strongly indicated that depletion of SRSF2 appeared to activate p21‐dependent pathway in primary myoblasts.

Previous reports demonstrated that SRSF2 functions as a potent transcription activator.^[^ ^15^, ^21^ ^]^ We then took advantage of public database and searched SRSF2 ChIP‐seq signals on the downregulated genes revealed by scRNA‐seq analysis. As expected, cell cycle‐involved genes *E2F1*, *Hjurp*, *Aurka*, and *Aurkb*, cytoskeleton‐related genes *Col1a1* and *Vimentin* showed strong SRSF2 binding signals on their promoter regions. And ChIP‐qPCR analysis and luciferase activity assays provided further evidence that SRSF2 directly regulated the expression of genes *Hjurp* and *E2F1* (Figure S14, Supporting Information). Hjurp has been identified as a key factor to promote chromosomal segregation and cell mitosis.^[^ ^22^ ^]^ Interestingly, knockdown of Hjurp significantly decreased myoblast proliferation and also increased p21 levels, similar to the SRSF2 knockdown (Figure S15, Supporting Information).

### 2.9 Mesen Cells Subcluster into Distinct Cell Populations

Skeletal muscle contains a complex arrangement of connective tissue, which are important for skeletal muscle development.^[^ ^23^ ^]^ Next, we performed unsupervised clustering on the Mesen group and observed further heterogeneity with 11 subclusters, which display distinct proportions of cell populations between Het/tdT and MKO/tdT samples (Figure S16A,B, Supporting Information). As shown, Mesen_1 subcluster displayed high expression of *Thbs4*, *Col3a1*, *Tnmd*, *Postn*, and *Cqgtnf3* (Figure S16C,D, Supporting Information). Expression of these genes were mainly enriched in the endomysium, perimysium and epimysium cells of skeletal muscle.^[^ ^23^ ^]^ Thus, we identified the Mesen_1 subcluster as skeletal muscle fibroblasts. On the other hand, we designed the Mesen_3 subcluster as dermal fibroblasts because *Foxd1* was enriched, and *Foxd1*‐expressing progenitors contributed to dermal fibroblast population.^[^ ^24^ ^]^ It is clear that loss of SRSF2 forced mesenchymal cell progenitors to change cell fate from skeletal muscle fibroblasts to the dermal fibroblasts. Moreover, differential gene expression analysis further revealed that expression of major components of extracellular matrix (ECM) *Postn*, *CD44*, *Ndn*, and *Col4a1* were significantly decreased in the Mesen_1 subcluster, indicating that the functional ECM production was severely damaged in the mutant muscles.

## 3 Discussion

Skeletal muscle formation is a highly precise and coordinated process, which requires the proliferation of myoblasts, permanent withdrawal from the cell cycle, differentiation and final fusion into multinucleated muscle fibers.^[^ ^25^ ^]^ Cell cycle arrest is a prerequisite for differentiation.^[^ ^26^ ^]^ The abnormal myogenesis observed in SRSF2‐null embryos could be the consequences of abnormal apoptosis and proliferative defects of MPCs, premature differentiation of myoblasts, and altered myogenic fate of Myf5‐derived cells.

Examination of Myf5‐Cre//Rosa26lacZ mice revealed the broad presence of Myf5‐derived cells in tissues originating from the paraxial mesoderm and the neuroectoderm.^[^ ^27^ ^]^ Consistent with this, our combined lineage tracing and scRNA‐seq analysis identified many previously unrecognized cell types that transiently harbor *Myf5* activity. These results could help explain some of previously reported discrepancies. For example, rib defects were initially reported in homozygous Myf5 mutant mice,^[^ ^5^, ^8^ ^]^ and later in mice with ablated Myf5‐expessing cells,^[^ ^27^ ^]^ but it is still controversial whether Myf5 was directly involved in the generation of the rib phenotype. Our data showed that the transient expression of Myf5 occurs in the progenitors of developmental chondrocytes and osteocytes. Loss of temporal Myf5 activity during the early development could be responsible for these rib defects, which are another hallmark features observed in the SRSF2‐null mice. In addition, Myf5‐dervied cells also contributed to mesenchymal cells, endothelial cells, pericytes, neural cells and Schwann cells. These non‐myogenic cell lineages could potentially play important roles during skeletal muscle development.

High rates of apoptosis and severe skeletal muscle defects were observed in the mutant mice, which is reminiscent of Rb mice.^[^ ^11^ ^]^ Interestingly, SRSF2 knockdown switched off E2F1‐dependent genes, while activated MyoD‐dependent genes, including increased pRb and decreased ppRb. This indicated a possibility that SRSF2 might control myogenesis through regulating cell‐cycle‐related genes. Significantly, scRNA‐seq analysis and immunostaining assay demonstrated that loss of SRSF2 resulted in precocious differentiation and a progressive depletion of the progenitor pool, as well as apoptosis. Moreover, p21 knockdown could rescue apoptosis and premature differentiation defects seen in SRSF2‐knockdown myoblasts. Fully in line with previous reports that SRSF2 functions as a potent transcription regulator,^[^ ^15^, ^21^ ^]^ strong SRSF2 binding signals were observed on the promoter regions of cell cycle‐involved genes *E2F1*, *Hjurp*, *Aurka*, and *Aurkb*, and cytoskeleton‐related genes *Col1a1* and *Vimentin*. Interestingly, we found that there were up to nine potential E‐box sequences within the promoter region of E2F1 and at least one E‐box for Vimentin (Figure S17, Supporting information). Therefore, it is possible that SRSF2 might regulate transcription by forming complex with MRFs during myogenesis, which need detailed investigation in the future.

It is well‐recognized that Pax3/Pax7 positive cells constitute a reserve of myogenic progenitors present not just in the mature myotome, but also in all developing muscle masses. And during the limb muscle development, Pax3^+^/Pax7^+^ MPCs give rise to cells that express Myf5 and/or MyoD. However, scRNA‐seq analysis revealed that Pax7 and Myf5 had a similar expression pattern in the Myf5‐derived proliferative myoblasts, whereas MyoD was wildly expressed in differentiated and proliferative myoblasts, and Pax3 signals were only weakly detected (Figure 5C). Pax3 was weakly expressed because Myf5 genetically downstream of Pax3 in the myogenic context,^[^ ^28^ ^]^ or because there were two distinct myogenic pathways defined by Pax3 and Myf5, respectively.^[^ ^6^ ^]^ These results indicated a seemly different genetic hierarchy than previously described exists in vivo myogenesis. Further investigation is required for verification.

Community effect was first described by Gurdon et al, which indicates that during skeletal muscle development, MPCs and myoblasts are required to be arranged as dense clusters in order to promote cell differentiation in union.^[^ ^18^ ^]^ In this study, we observed the community effect was totally lost in the SRSF2‐null mice. Myf5‐derived cells in the absence of SRSF2 appeared to move randomly in the limb, and some even migrated into the livers and bones. This randomly dispersed pattern was companied by drastically impaired myogenesis and exhaustion of MPCs in the mutant mice. scRNA‐seq analysis revealed that almost 50% of Myf5‐drived cells contributed to the Mesen population, and subclustering analysis of this population confirmed that *Foxd1*‐enriched dermal fibroblasts^[^ ^24^ ^]^ were abundant in the mutant mice at the expense of *Thbs4*/*Col3a1*‐enriched skeletal muscle fibroblasts. These findings indicated that loss of SRSF2 also severely impaired development of mesenchymal cells surrounding muscle cells. And the destruction of skeletal muscle cell microenvironment could lead to loss of community effect in the knockout mice. This was totally consistent with previous reports that some unknown factors in the limb mesenchyme would act in providing the community effect to promote proliferation and differentiation of limb muscle cells.^[^ ^29^ ^]^

## 4 Conclusion

In conclusion, our data demonstrated thatMyf5‐derived cells contributed to various cell lineages than previously described, and SRSF2 was a critical regulator for embryonic myogenesis. SRSF2 regulates the entry of Myf5 cells into the myogenic program and ensures their survival by preventing precocious differentiation and apoptosis.

## 5 Experimental Section

### Generation of Mutant Animals

SRSF2^f/f^ mice were generously provided by Dr. Fu. The SRSF2^f/f^ mice were crossed with transgenic C57BL/6J mice expressing Cre recombinase driven by the Myf5 promoter. Resulting progeny (SRSF2^f/w^/Myf5‐Cre) were mated with SRSF2^f/f^ mice to generate skeletal muscle‐specific SRSF2 knockout mice SRSF2^f/f^/Myf5‐Cre (MKO). The Rosa26‐LSL‐tdtomato (hereafter tdT) mice were purchased from The Jackson Laboratory and have been described previously.^[^ ^14^ ^]^ The SRSF2^f/f^ mice were crossed with dT breeders to generate SRSF2^f/f^/tdT mice. Next, SRSF2^f/f^/tdT mice were crossed with SRSF2^f/w^/Myf5‐Cre mice to generate SRSF2^f/f^/Myf5‐Cre/tdT mice (MKO/tdT). SRSF2^f/w^/Myf5‐Cre/tdT(Het/tdT) mice served as controls. Primer sequences used in the genotyping are listed in Table S3, Supporting Information. All experiments were conducted in accordance with the guidelines of the Institutional Animal Care and Use Committee of Shanghai Institute for Nutrition and Health Nutritional Sciences, Chinese Academy of Sciences.

### Primary Myoblast Isolation, Cell Culture, Differentiation, and Transfection

Primary myoblasts were isolated from the dorsal muscle and limbs from E16 to E16.5 embryos. Briefly, muscles were isolated, minced, and dissociated in 0.2% (wt/vol) collagenase‐type XI (Sigma‐Aldrich) and 2.4 U mL^−1^ dispase II (Invitrogen) in DMEM (Gibco) at 37 ℃. After centrifugation, the pellet was filtered through an 80 µm cell strainer and resuspended in growth medium containing F‐10 (ThermoFisher) supplemented with 20% FBS (Gibco), 2.5 ng mL^−1^ FGF, 1% penicillin/streptomycin. Cells were laid repeatedly on the dish for 3 h to remove the adherent cells, then unattached cells (myoblasts) were harvested and plated on the Matrigel‐coated (1% v/v Matrigel in DMEM) dishes. Cells were not allowed to subculture 75% confluence during the passage. Primary myoblasts were incubated in growth medium containing F‐10 supplemented with 20% FBS, 2.5 ng mL^−1^ FGF, 1% penicillin/streptomycin. C2C12 cells were incubated in growth medium containing DMEM supplemented with 10% FBS and 1% penicillin/streptomycin. Myogenic differentiation of both primary cells and C2C12 cells was activated by replacing the growth medium with a differentiation medium (DMEM supplemented with 2% horse serum). For transient transfection, primary myoblasts and C2C12 cells were transfected with siRNA or plasmids using the Lipofectamine RNAiMAX (Invitrogen) or Lipofectamine 200 (Invitrogen) as described in manufacturer's protocol. The siRNA sequences used are listed in Table S6, Supporting Information.

### RNA Extraction, cDNA Synthesis, and qPCR

RNA extraction, cDNA synthesis and qPCR were conducted as previously described.^[^ ^30^ ^]^ Total RNA was extracted from mouse tissue or cultured cells with Trizol Reagent (Invitrogen). cDNA was synthesized using the High‐Capacity cDNA Reverse Transcription kit (Applied Biosystems). Gene expression was assessed using standard qPCR approaches with SYBR Premix Ex Taq kit (TaKaRa). Analysis was performed on a QuantStudio Real‐Time PCR System (Applied Biosystems). Data was normalized relative to Rplp0. Primer sequences used in qPCR are listed in Table S2, Supporting Information.

### WB Analysis

Cells were homogenized in RIPA Lysis and Extraction Buffer (Thermo Scientific) supplemented with Complete Protease Inhibitor Cocktail (Roche). Equal amounts of proteins were separated on SDS‐PAGE gels, then transferred to immobilon‐NC Transfer Membrane (Millipore). Membranes were incubated with primary antibodies overnight and then with HRP‐conjugated secondary antibodies for 1 h. Specific signals were detected with the chemiluminescence system (Tanon‐4100). The detailed information of antibodies used is listed in Table S1, Supporting Information.

### HE, TUNEL Assays, and Immunostaining

HE and TUNEL assay on the paraffin sections were performed using a standard protocol. 6 µm‐thick sections of tissues were processed for HE staining. 5 µm‐ thick sections were processed for TUNEL staining according to the manufacturer's protocol (Promega, catalog no. G3250). Immunofluorescence on the paraffin tissues and frozen sections was conducted as previously described.^[^ ^30^, ^31^ ^]^ Briefly, 6 µm paraffin tissues were deparaffinized in xylol and rehydrated with gradient ethanol followed by antigenic retrieval. Both frozen and paraffin sections were blocked with 5% goat serum (Santa Cruz Biotechnology) and then incubated with primary antibodies overnight at 4 ℃, followed by incubation with secondary antibodies as well as DAPI for 1 h. For cellular immunostaining, cells were fixed in 4% paraformaldehyde for 15 min, permeabilized with 0.2% Triton X‐100 in PBS and blocked with 5% goat serum, followed by incubation with primary antibodies overnight at 4 ℃ and secondary antibodies as well as DAPI for 1 h. Immunofluorescence images were acquired on a Zeiss LSM880NLO Confocal Microscope. Quantifications were performed using ImageJ software (National Institutes of Health). The detailed information of used antibodies is listed in Table S1, Supporting Information.

### Chromatin Immunoprecipitation Assays (ChIP‐qPCR)

Potential SRSF2 binding sites located on the Hjurp and E2F1 promoter regions were obtained from the Gene Expression Omnibus (GEO) under the accession number GSM1106075 and GSM1106077. Based on this, two pair of primers were designed for the ChIP‐qPCR analysis. Myoblasts were transiently transfected with SRSF2‐HA and vector‐HA plasmids for 48 h. Then ChIP was performed with Magna ChIP kit (Millpore; catalog no. MAGNA0017) according to the manufacturer's instructions, using anti‐HA antibodies (Abcam) for IP with normal mouse IgG as negative control. The immunoprecipitated DNA and input DNA were quantified by qPCR analysis. The detailed information of antibodies used is listed in Table S1, Supporting Information. Primer sequences used in ChIP‐qPCR are listed in Table S4, Supporting Information.

### Dual‐Luciferase Activity Assay

For construction of luciferase reporter plasmids, the Hjurp promoter region (position −536 bp to 522 bp from the transcription start site, TSS) or E2F1 promoter region (position −680 bp to 767 bp from TSS) was inserted into the pGL3 vector (Promega). 293T cells were transfected using polyethylenimine (PEI; Sigma) according to the standard conditions with a 1:1 ratio of DNA (mg) and PEI (ml). For reporter assays, 293T cells were transfected with pRL‐TK plasmid and Hjurp‐luciferase plasmid or E2F1‐Luciferase plasmid, plus vector‐HA or SRSF2‐HA. Cells extracts were harvested post 48 h transfection. Dual‐luciferase assays were performed according to the manufacturer's instructions with a Dual‐Luciferase assay kit (Promega). The primer sequences used for pGL3‐based reporter plasmid construction are listed in Table S5, Supporting information.

### Cell Viability Assays

For cell viability assay, 1 × 10^3^ C2C12 cells were plated in 96‐well plates, and transfected with vector‐HA or SRSF2‐HA. The proliferation rate of cells was detected using the CCK8 method based on the manufacturer's instructions. Cell viability was assessed at 1, 2, 3 and 4 days after transfection.

### Preparation of Single Cell Suspension and scRNA‐seq

E14 MKO/tdT and Het/tdT embryos were collected, decapitated and eviscerated. To generate single cells, truncated embryos were incubated in 0.2% (wt/vol) collagenase‐type XI (Sigma‐Aldrich) and 2.4 U mL^−1^ dispase II (Invitrogen) in DMEM at 37 ℃ 30 min, followed by filtering through a 40‐µm cell strainer. Cells were then centrifuged, resuspended in cold 5% FBS in PBS, and immediately sorted with a Beckman moflo Astrios EQ FACS sorter. Endogenous tdT signal was detected through PE channel and gates were set up according to the positive and negative controls. Sorted tdT+ cells were resuspended in 20% FBS/PBS buffer and loaded onto a droplet‐based library prep platform Chromium (10x Genomics) with a Chromium Single Cell Reagent Kit (version 3) according to the manuscript's instructions. Sequencing was performed on an Illumina nova 6000 and 150‐base pair paired‐end reads. The sequencing and bioinformatics analysis were performed by OE Biotech Co., Ltd. (Shanghai, China). All statistical analyses, unless otherwise specified, were conducted using R.^[^ ^32^ ^]^

### scRNA‐seq Data Preprocessing

Raw sequencing data was processed by Cell Ranger software suite (10x genomics, version 5.0.0) including demultiplexing cellular barcodes, mapping reads to the reference, and down‐sampling reads to aggregate data from samples, resulting in a gene count matrix. Next cells of low‐quality or likely multiplets were removed if the unique molecular identifier (UMI)/gene counts were two standard deviations (SD) from the mean of all cells. Cells with >10% of the counts from mitochondrial genes were further filtered out. *NormalizedData* function from Seurat (version 3.1.1) was then applied to cells to normalize and log transform the data with the option “LogNormalize”. To reduce the dimensionality of the data, first, top variable genes were selected using *FindVariableGenes* function in Seurat with *mean.function* = “FastExpMean” and *dispersion.funciton* = “FastLogVMR”, and then performed Principal component analysis (PCA) with *RunPCA* function. Graph‐based clusters were identified using the *FindClusters* function and visualized by t‐distributed stochastic neighbor embedding (t‐SNE) plot using *RunTSNE* and *DimPlot* functions in Seurat. Marker genes for each cluster were identified by the *FindAllMarkers* function (test.use = bimod) by comparing the gene expression in the cluster of interest versus all other clusters. Differentially expressed genes (DEGs) were idenfied using the *FindMarkers* function (test.use = MAST) with *p* value < 0.05 and |log2foldchange| > 0.58. Gene Ontology (GO) and Kyoto Encyclopedia of Genes and Genomes (KEGG) pathway enrichment analysis of DEGs were respectively tested assuming a hypergeometric distribution.

### Pseudotime Analysis

The Monocle2 package^[^ ^19^ ^]^ was used to study the developmental pseudotime. Raw counts in Seurat object were first converted to CellDataSet object by the *importCDS* function in Monocle2. Then genes which were informative in ordering cells along the pseudotime trajectory were identified by the *differentialGeneTest* (qval < 0.01). The dimensional reduction clustering analysis was performed with the *reduceDimension* function, followed by trajectory inference with the *orderCells* function using default parameters. The *plot_genes_in_pseudotime* function was applied to track gene expression and to track changes over pseudotime. To discover the order of gene‐expression and functional events during cell state transitions at a single‐cell resolution, GeneSwitches analyses were conducted. Specifically, gene expression data was first binarized into 1 (on) or 0 (off) state with the *binarize_exp* function in the GeneSwitches package (fix_cutoff = TRUE, binarize_cutoff = 0.05). A gaussian noise with mean of 0 and s.d. of 0.1 added to the gene expression data, which ensures numerical stability in the model fitting. Genes were also filtered without a distinct bimodal “on ‐off” distribution. A logistic regression model was then fitted to model the binary states (on or off) by the *find_switch_logistic_fastglm* function with random downsampling of zero expressions (downsample = TRUE) to adjurst genes with high zero inflation. Finally, the top 50 best fitting genes (high McFadden's Pseudo R^2^) were plotted along the pseudo‐timeline with “switched on” above the line, and “switched off” below the line.

### Data Availability

The data sets have been deposited in the GEO database under the accession number GSE189088.

### Statistical Analyses

Statistical analysis was performed by GraphPad Prism 8.0 software. All of the data shown in the histograms were the results of at least three independent experiments and presented as the mean ± standard deviation (SD). The sample size (*n*) for each statistical analysis was indicated in detail in individual figure legend. Differences between groups were tested for statistical significance by using the two‐tailed unpaired Student's *t*‐test. Differences between values were considered statistically significant when * *p* < 0.05, ** *p* < 0.01, and *** *p* < 0.001.

## Conflict of Interest

The authors declare no conflict of interest.

## Supporting information

Supporting Information

## Data Availability

The data that support the findings of this study are openly available in GEO at https://www.ncbi.nlm.nih.gov/geo/, reference number 189088.
