# Supplementary material for: Single‐Cell RNA Sequencing Reveals Heterogeneity of Myf5‐Derived Cells and Altered Myogenic Fate in the Absence of SRSF2
Source: Adv Sci (Weinh). 2022 Apr 23;9(18):2105775. doi: 10.1002/advs.202105775 (PMC9218650; doi:10.1002/advs.202105775)
Supplement: Supplementary file 1 — Supporting Information [file ADVS-9-2105775-s001.pdf]

ADVANCED SCIENCE
Open Access

Supporting Information

for *Adv. Sci.*, DOI 10.1002/advs.202105775

Single-Cell RNA Sequencing Reveals Heterogeneity of Myf5-Derived Cells and Altered Myogenic Fate in the Absence of SRSF2

Ruochen Guo, Xue You, Kai Meng, Rula Sha, Zhenzhen Wang, Ningyang Yuan, Qian Peng, Zhigang Li, Zhiqin Xie, Ruijiao Chen and Ying Feng**

Supplementary Information

Single-cell RNA sequencing reveals heterogeneity of Myf5-derived cells and altered myogenic fate in the absence of SRSF2

Ruochen Guo, Xue You, Kai Meng, Rula Sha, Zhenzhen Wang, Ningyang Yuan, Zhigang Li, Zhiqin Xie, Ruijiao Chen and Ying Feng**

This file includes:

Supplementary Figure S1 to S17

Table S1 to S6

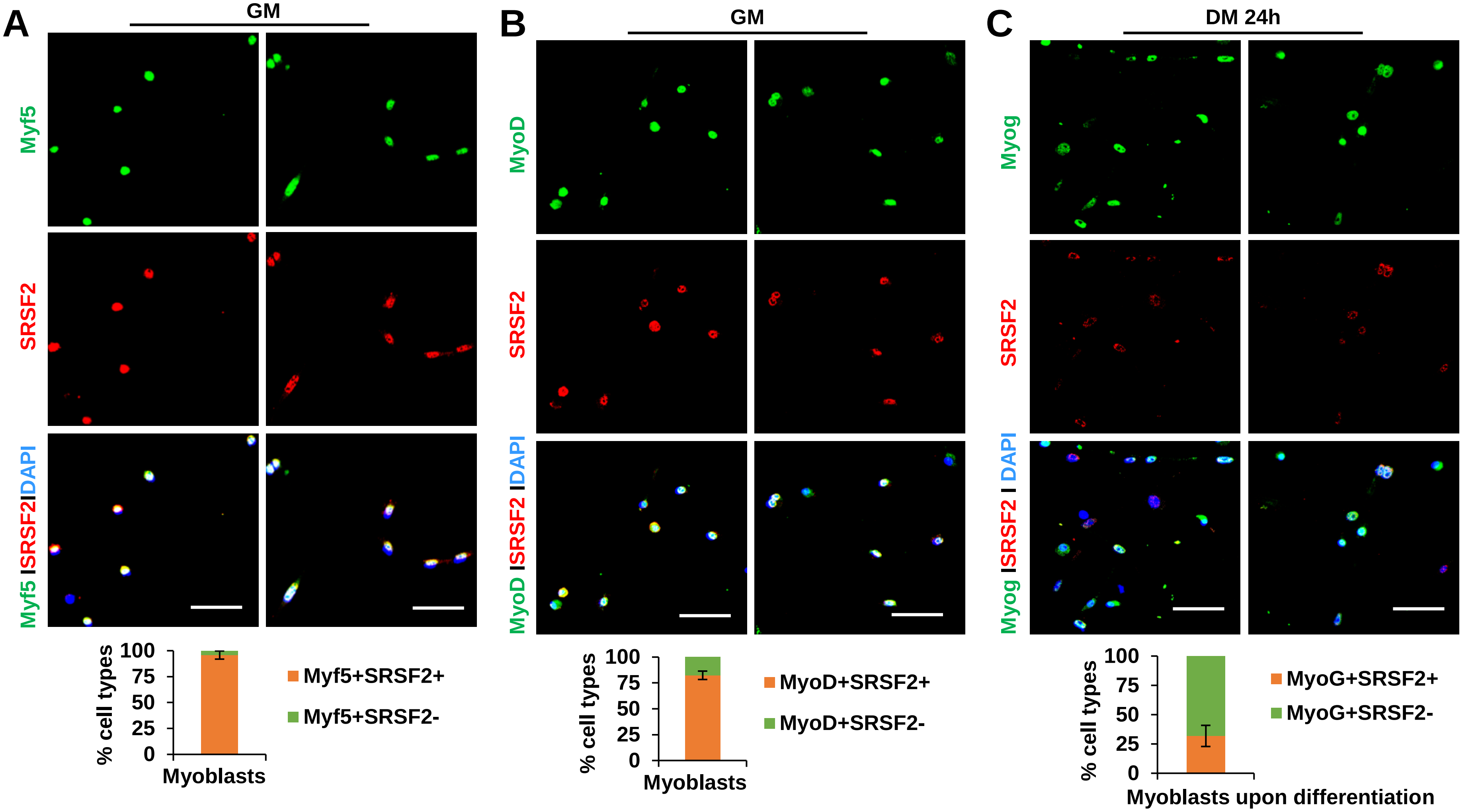

Figure S1. Immunostaining of SRSF2 and MRFs in primary myoblasts. A-B) Representative confocal images of Myf5 (green) and SRSF2 (red) colocalization, and MyoD (green) and SRSF2 (red) colocalization in primary myoblasts incubated with growth medium (GM). Scale bars, 50 μm. 1x10^3 cells were placed on the coverslips for staining. The mean percentage of Myf5 and SRSF2 colocalization, and MyoD and SRSF2 colocalization per area are shown on the below bar graphs (n = 3). Data are shown as the mean ± SD. C) Representative confocal images of Myog (green) and SRSF2 (red) colocalization upon myoblast differentiation. Scale bars, 50 μm. The mean percentage of Myog and SRSF2 colocalization per area are shown on the below bar graph (n = 3). Data are shown as the mean ± SD.

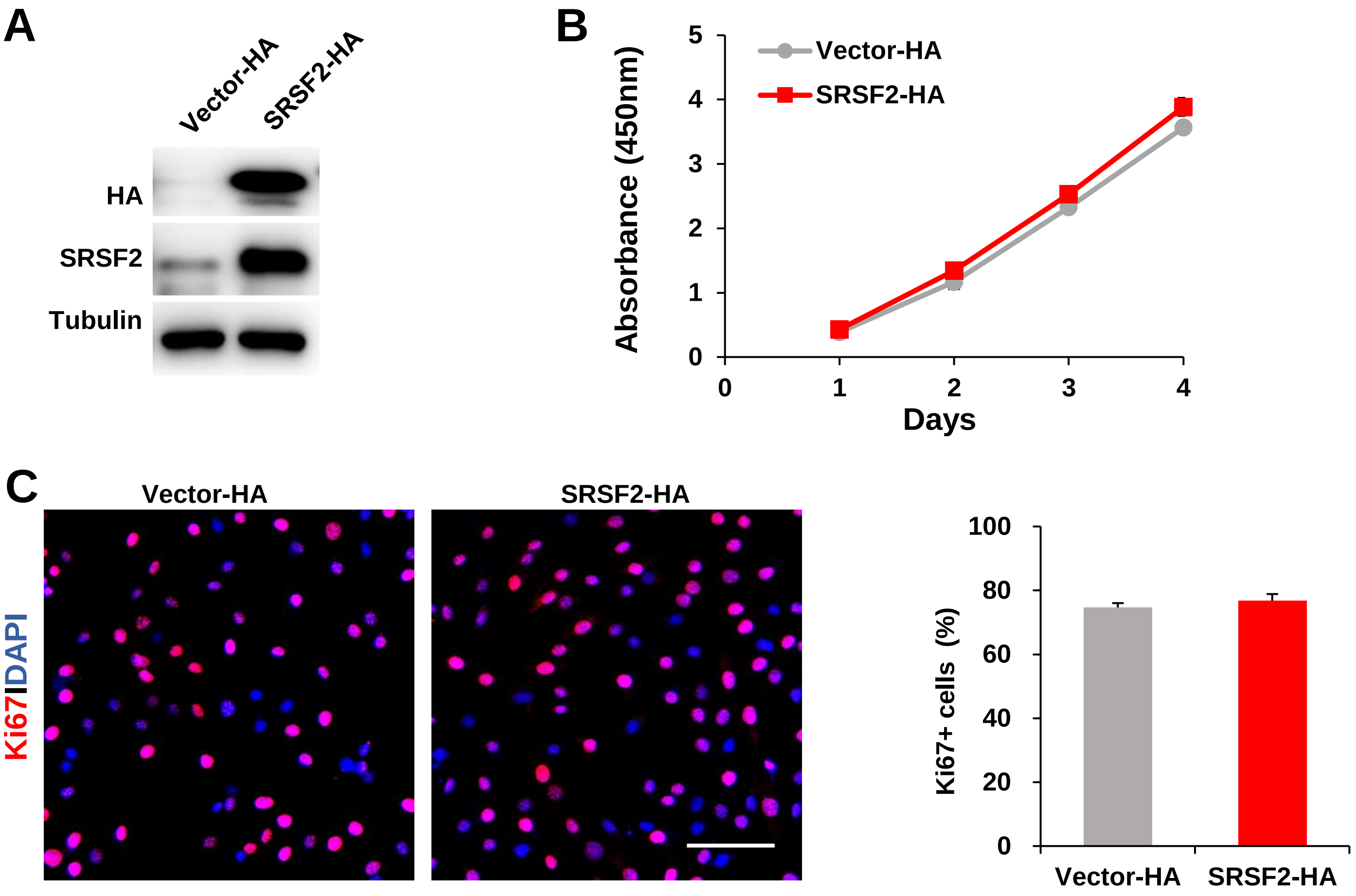

Figure S2. Overexpression of SRSF2 has modest effects on C2C12 cell proliferation. A) Representative WB analysis of HA, SRSF2 in C2C12 cells after 48h transfection with SRSF2 over-expression plasmids (SRSF2-HA) or control plasmid vector (Vector-HA) (n=3). Tubulin was used as the loading control. B) Growth curves of cells described in (A) assessed by CCK8 assay (n=3). The results were plotted as the mean ± SD. C) Representative confocal images of Ki67 (red) and DAPI (blue) immunostaining in C2C12 cells after 48h transfection (n=3). Scale bars, 100 μm. The percentage of Ki67 positive cells are shown on the right bar graph. Data are shown as the mean ± SD.

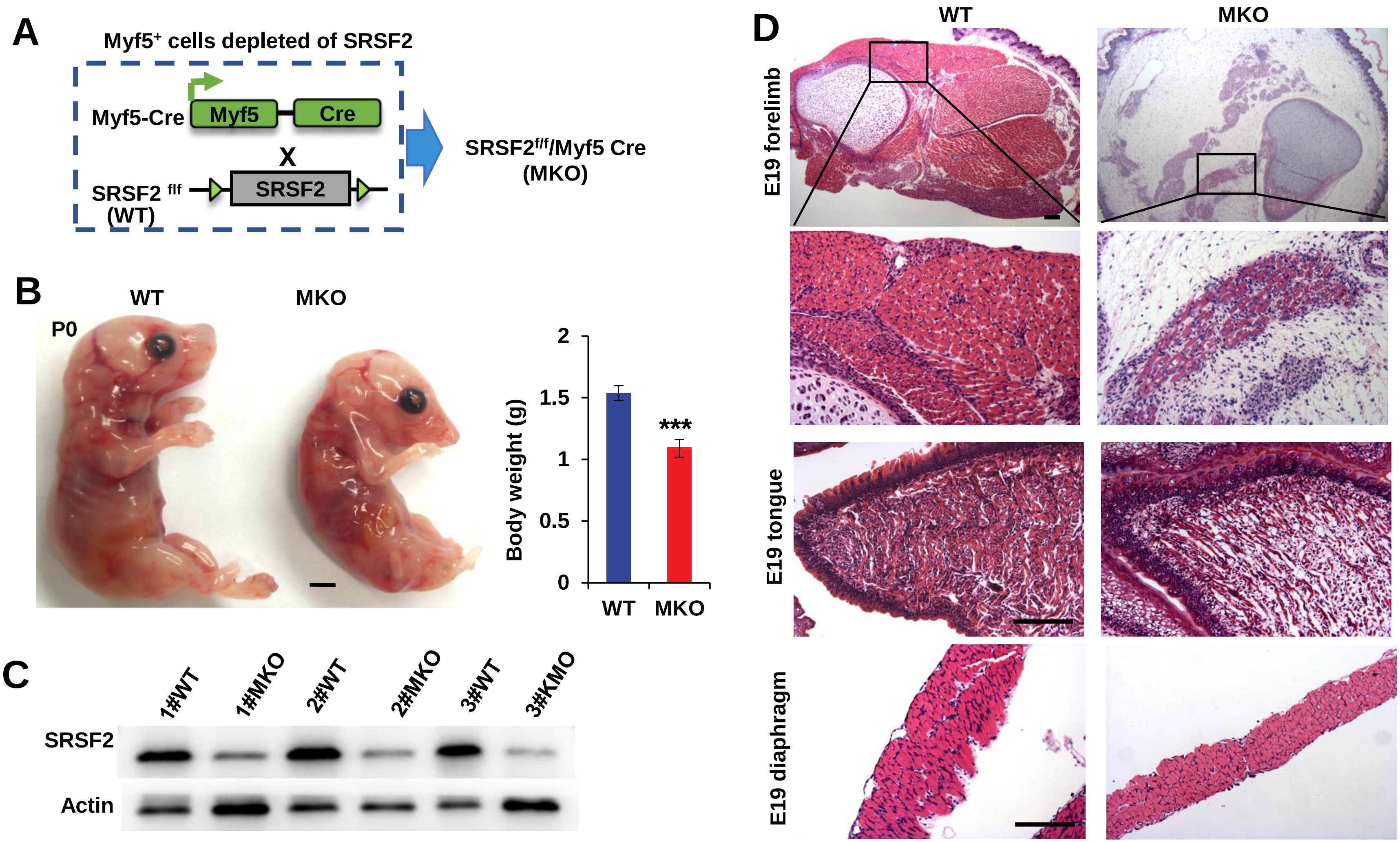

Figure S3. SRSF2 is essential for skeletal muscle development. A) Model of SRSF2$^{f/f}$ (WT) mice and knockout strategy was shown. B) Representative photos of WT and MKO mice are shown shortly after birth. Muscle defects and rib defects are easily observed in MKO mice after skin removal. Scale bars, 2 mm. Body weight was compared between WT mice and MKO mice shortly after birth (n = 5). Data represent mean ± SD. *** indicates P < 0.001, 2-tailed Student's *t-test*. C) Representative WB analysis of SRSF2 in hindlimbs of WT and MKO mice at E19 (n=3). Actin protein levels were used as loading controls. D) Representative hematoxylin and eosin (HE) staining of forelimb, tongue and diaphragm sections at E19 (n=5). Scale bar, 100 μm.

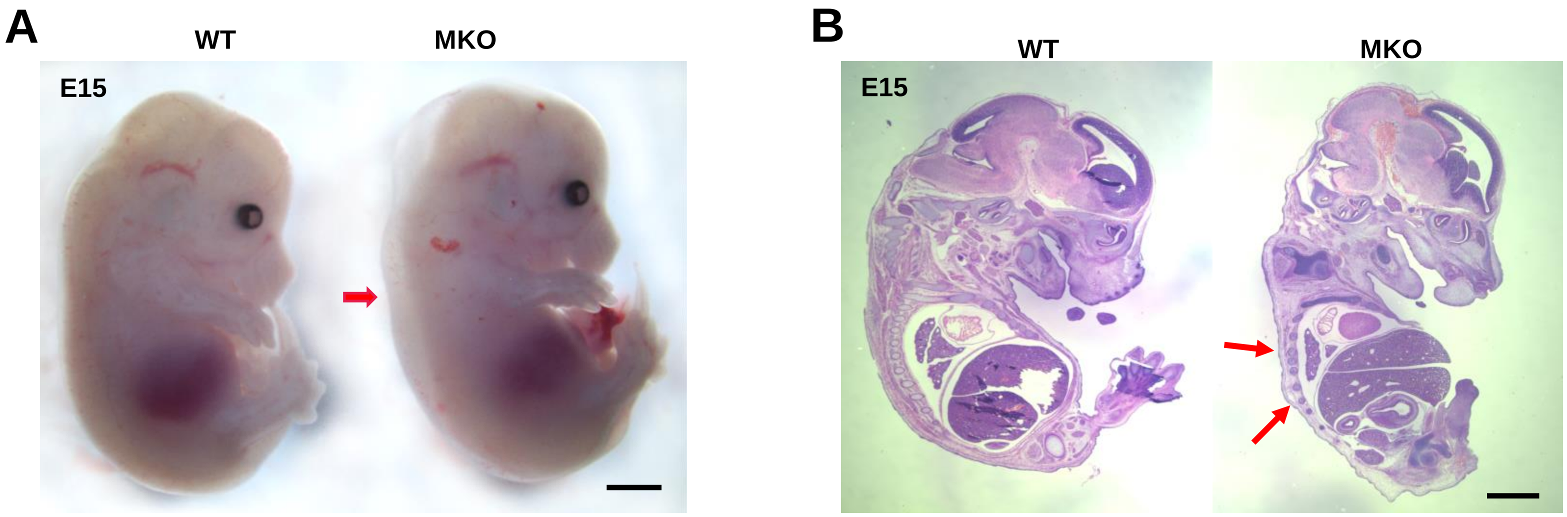

Figure S4. Mutant mice displayed severe edema and loss of back muscle at E15. A) Representative photos of WT and MKO embryos at E15 (n=3). Scale bars, 2 mm. The red arrow points to the edema in the mutant embryo. B) Representative HE staining of sections at E15 (n=3). Scale bars, 2 mm. Red arrows indicate severe muscle loss on the body wall.

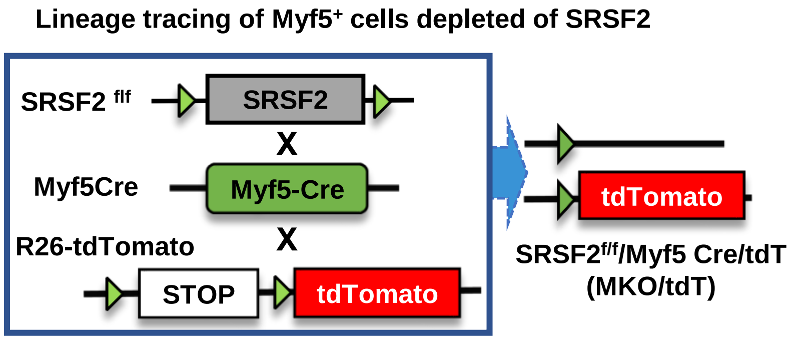

Figure S5. Model of SRSF2$^{f/f}$/Myf5 Cre/tdT mice lineage-tracing and knockout strategy. Schematic diagram of SRSF2$^{f/f}$/Myf5 Cre/tdT mice prepared by crossing SRSF2$^{f/f}$ mice with Myf5-Cre mice and with R26-tdTomato mice.

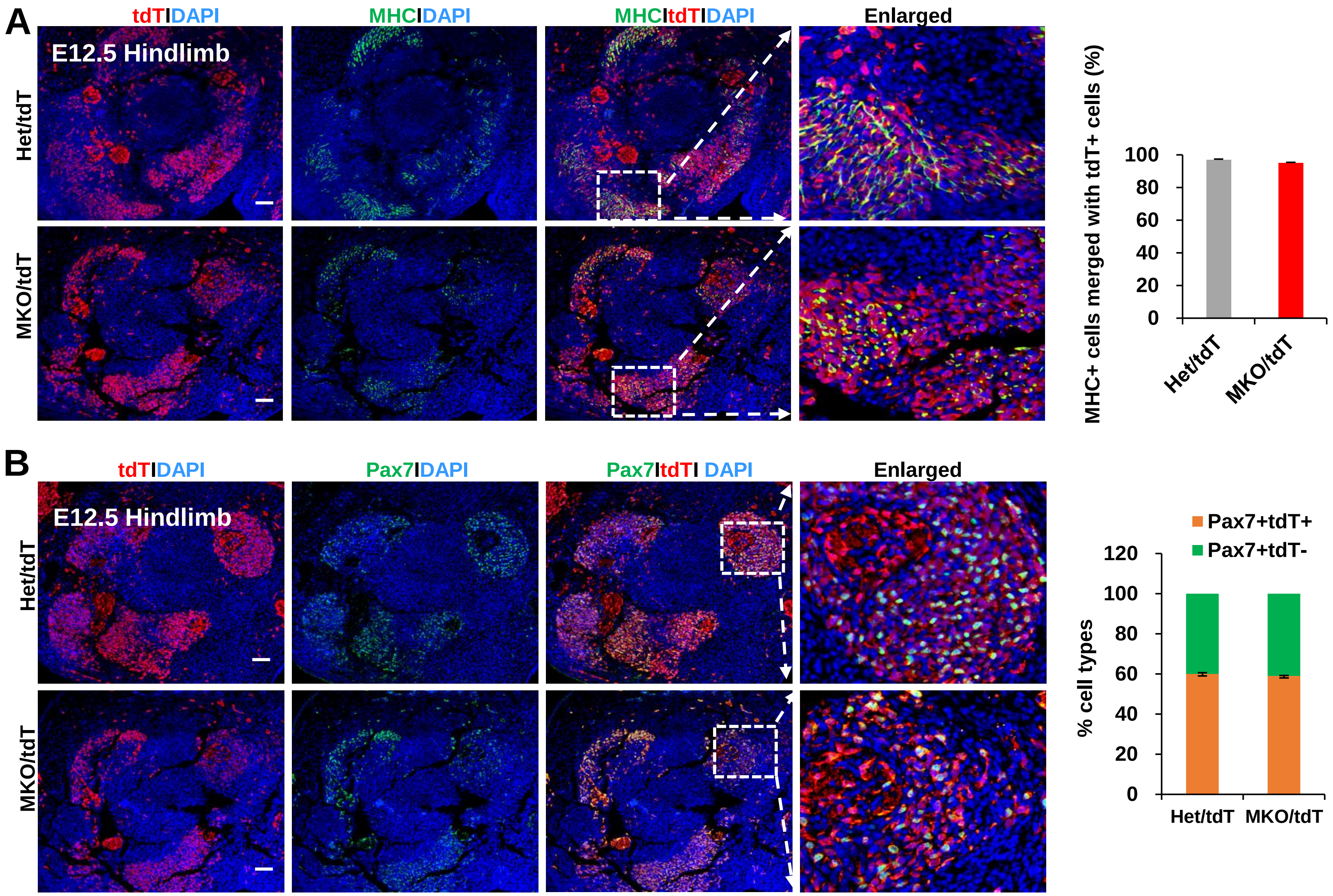

Figure S6. Relatively normal myogenesis was observed in mutant mice at E12.5. A-B) Representative confocal images of tdT (red), MHC (green), Pax7 (green) immunostaining in hindlimbs of Het/tdT and MKO/tdT embryos at E12.5. Scale bars, 100 μm. Note that white dotted boxes are shown enlarged in the right panels. Qualification was shown on the right panel bar graphs (n=3). All data are shown as the mean ± SD.

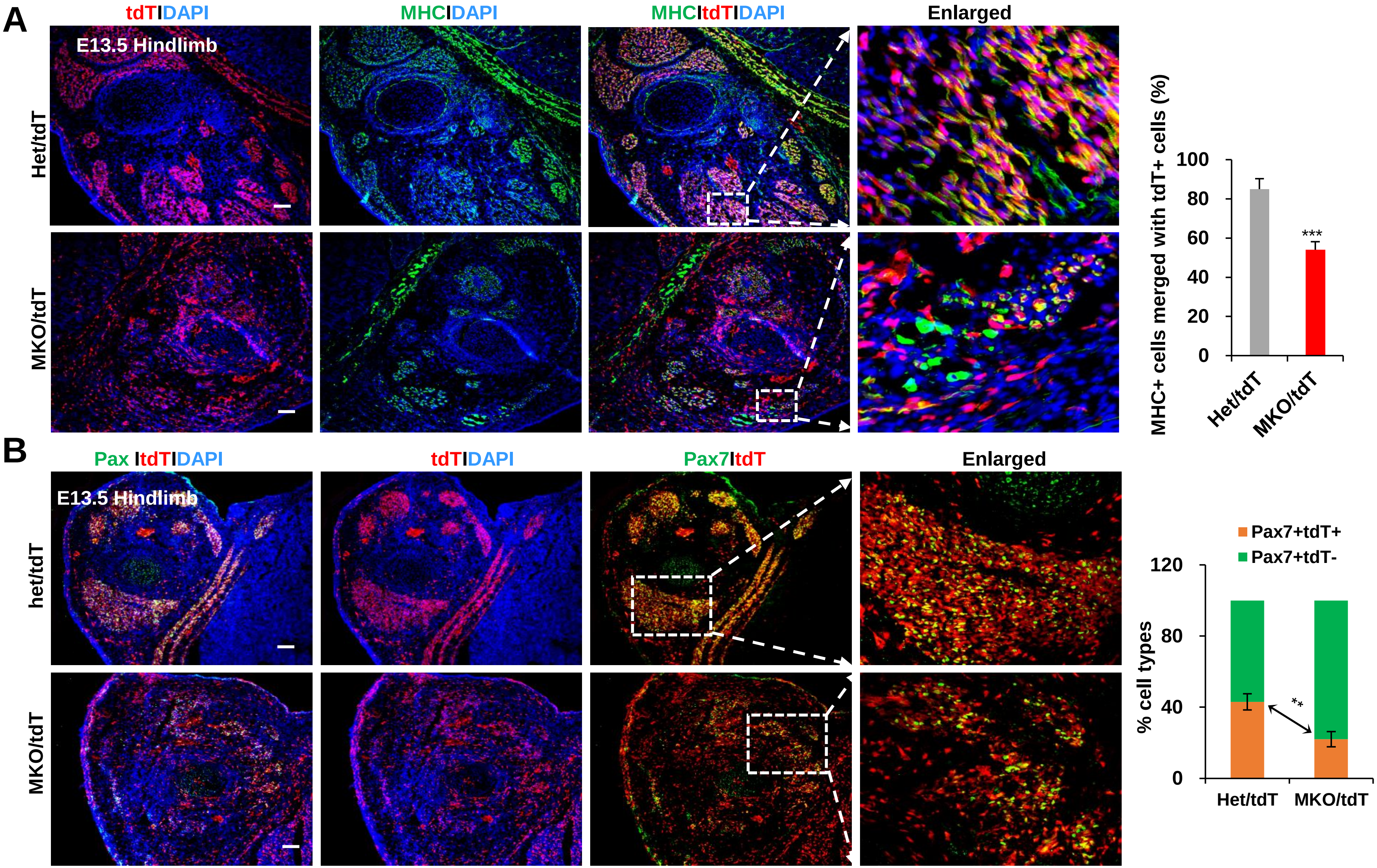

Figure S7. Abnormal myogenesis was observed in the mutant mice at E13.5. A-B) Representative confocal images of tdT (red), MHC (green) and Pax7 (green) immunostaining in hindlimbs of Het/tdT and MKO/tdT embryos at E13.5. Scale bars, 100 μm. Note that white dotted boxes are shown enlarged in the right panels. Qualification was shown on the right panel bar graphs (n=3). All data are shown as the mean ± SD. 2-tailed Student's t-test. **p < 0.01,*** p < 0.001.

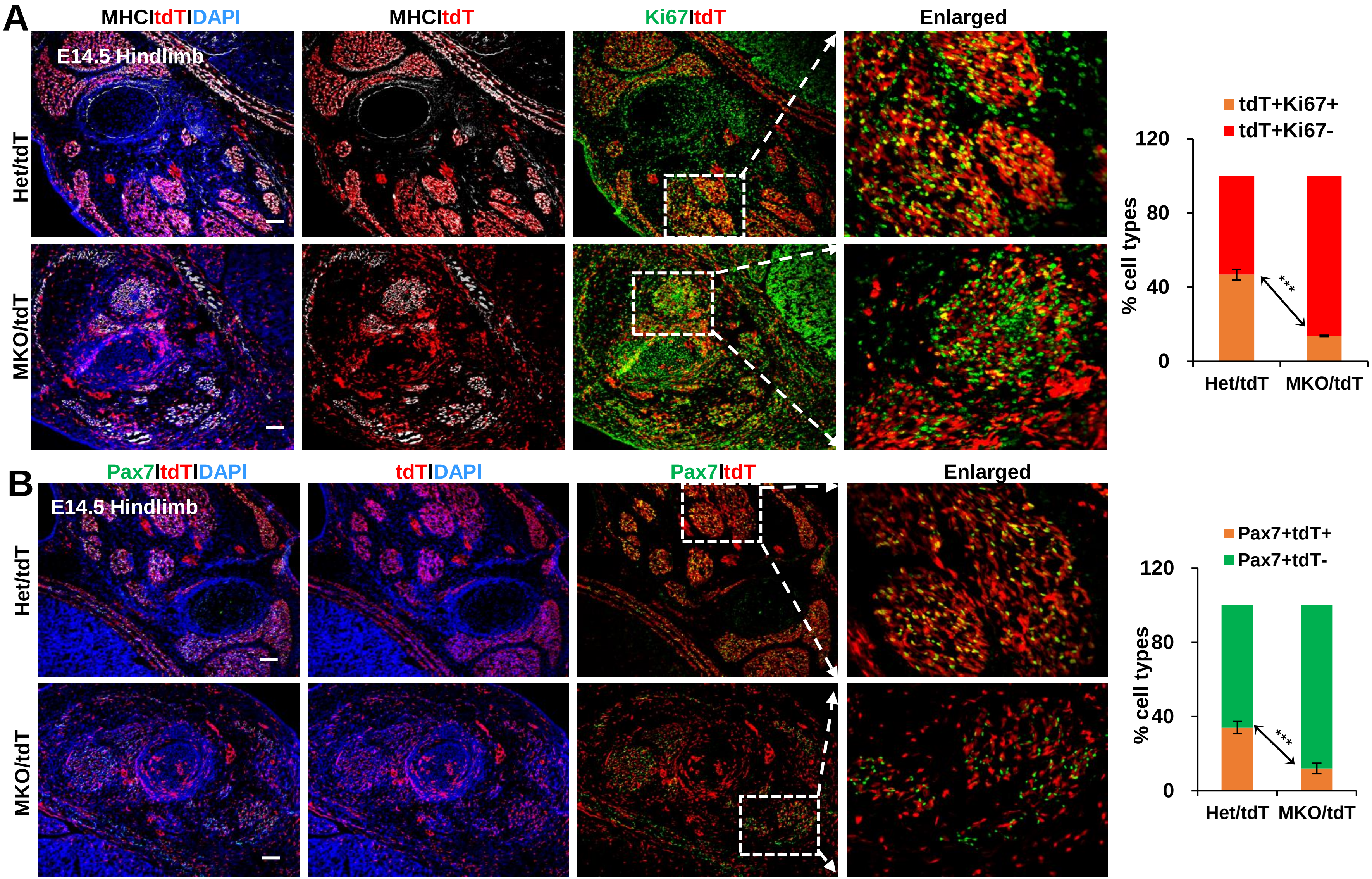

Figure S8. Abnormal myogenesis was observed in the mutant mice at E14.5. A-B) Representative confocal images of tdT (red), MHC (white), Ki67 (green), Pax7 (green) immunostaining in hindlimbs of Het/tdT and MKO/tdT embryos at E14.5. Scale bars, 100 μm. Note that white dotted boxes are shown enlarged in the right panels. Qualification was shown on the right panel bar graphs (n=3). All data are shown as the mean ± SD. 2-tailed Student's t-test.*** $p < 0.001$.

Het/tdT

15,268
Estimated Number of Cells

37,426	2,814
Mean Reads per Cell	Median Genes per Cell

Sequencing

Number of Reads	571,426,356
Number of Short Reads Skipped	0
Valid Barcodes	96.2%
Valid UMIs	99.9%
Sequencing Saturation	39.5%
Q30 Bases in Barcode	96.4%
Q30 Bases in RNA Read	89.0%
Q30 Bases in UMI	95.6%

Mapping

Reads Mapped to Genome	89.0%
Reads Mapped Confidently to Genome	86.4%
Reads Mapped Confidently to Intergenic Regions	4.7%
Reads Mapped Confidently to Intronic Regions	19.1%
Reads Mapped Confidently to Exonic Regions	62.7%
Reads Mapped Confidently to Transcriptome	58.0%
Reads Mapped Antisense to Gene	2.6%

Cells

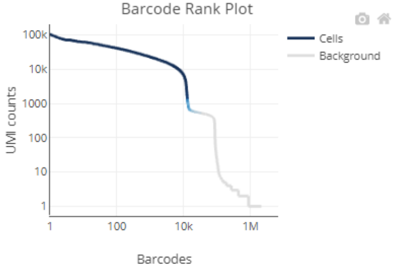

Estimated Number of Cells	15,268
Fraction Reads in Cells	80.9%
Mean Reads per Cell	37,426
Median Genes per Cell	2,814
Total Genes Detected	24,480
Median UMI Counts per Cell	9,219

Sample

Sample ID	WT
Chemistry	Single Cell 3' v3
Include introns	False
Reference Path	…nger-refdata/refdata-gex-mm10-2020-A
Transcriptome	mm10-2020-A
Pipeline Version	cellranger-5.0.0

MKO/tdT

12,498
Estimated Number of Cells

46,039	2,808
Mean Reads per Cell	Median Genes per Cell

Sequencing

Number of Reads	575,393,483
Number of Short Reads Skipped	0
Valid Barcodes	96.5%
Valid UMIs	99.9%
Sequencing Saturation	45.9%
Q30 Bases in Barcode	96.3%
Q30 Bases in RNA Read	89.6%
Q30 Bases in UMI	95.6%

Mapping

Reads Mapped to Genome	90.7%
Reads Mapped Confidently to Genome	88.1%
Reads Mapped Confidently to Intergenic Regions	4.9%
Reads Mapped Confidently to Intronic Regions	18.1%
Reads Mapped Confidently to Exonic Regions	65.1%
Reads Mapped Confidently to Transcriptome	60.8%
Reads Mapped Antisense to Gene	2.1%

Cells

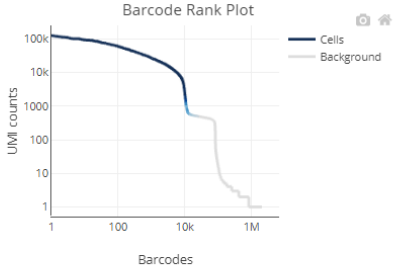

Estimated Number of Cells	12,498
Fraction Reads in Cells	81.3%
Mean Reads per Cell	46,039
Median Genes per Cell	2,808
Total Genes Detected	24,871
Median UMI Counts per Cell	9,372

Sample

Sample ID	KO
Chemistry	Single Cell 3' v3
Include introns	False
Reference Path	…nger-refdata/refdata-gex-mm10-2020-A
Transcriptome	mm10-2020-A
Pipeline Version	cellranger-5.0.0

Figure S9. Quality control metrics of the Het/tdT samples and MKO/tdT samples

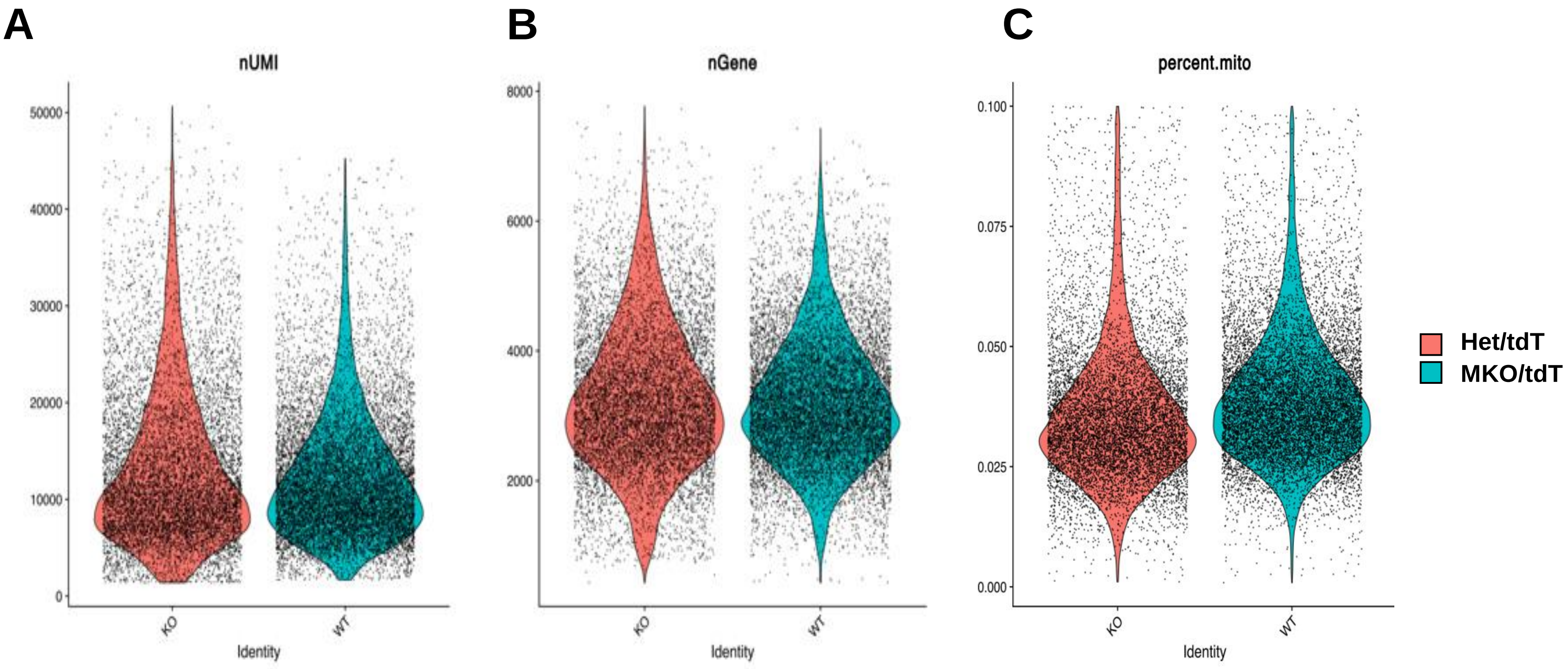

Figure S10. The distribution of the nUMI (A), nGene (B), and the percentage of mitochondrial counts (C) of the samples.

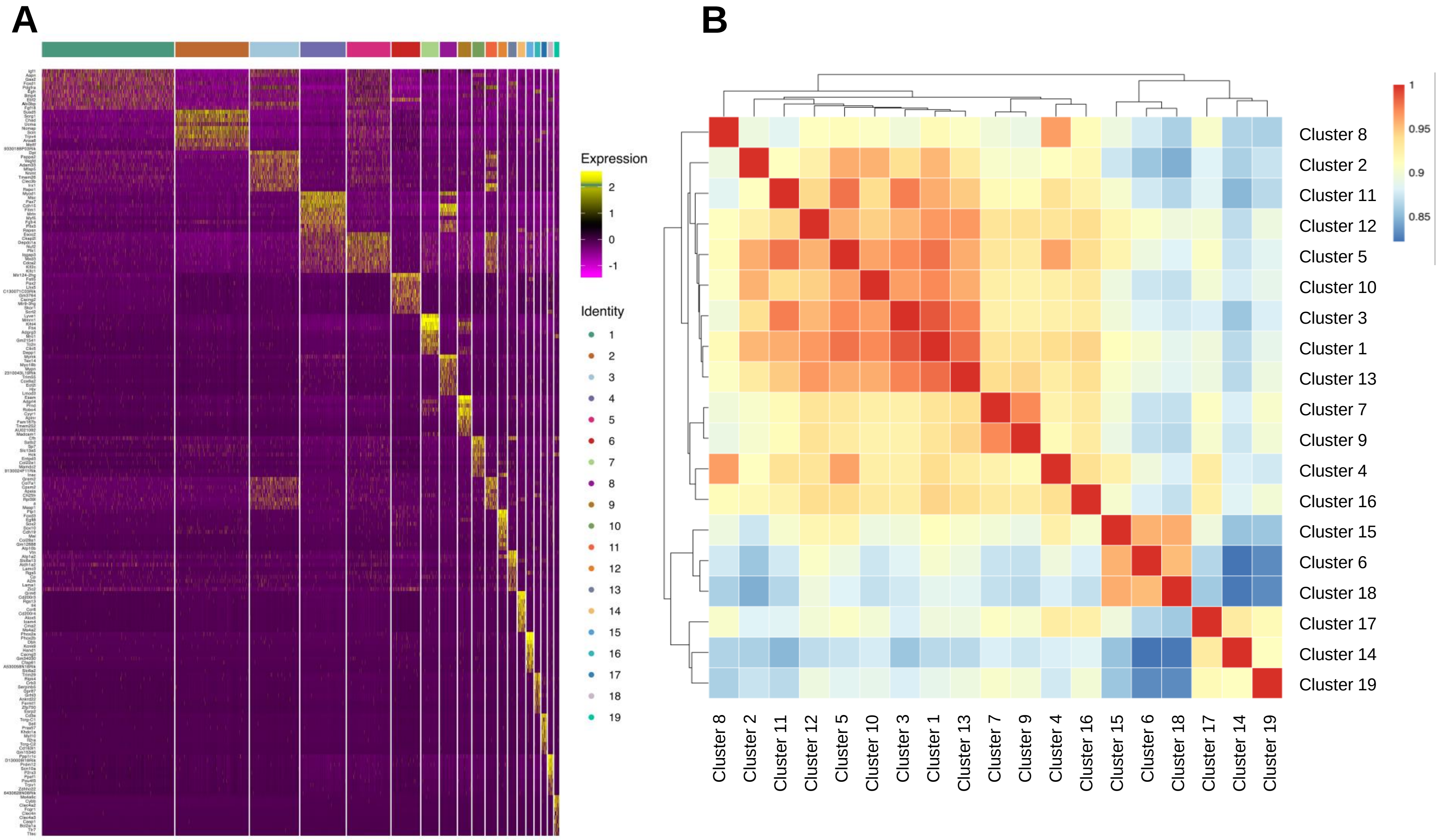

Figure S11. A) Heatmap representing the top 10 most differently expressed genes between cell clusters identified. Colour numbers correspond to the cell clusters shown in Figure 5B. B) The cluster-to-cluster distance of cells.

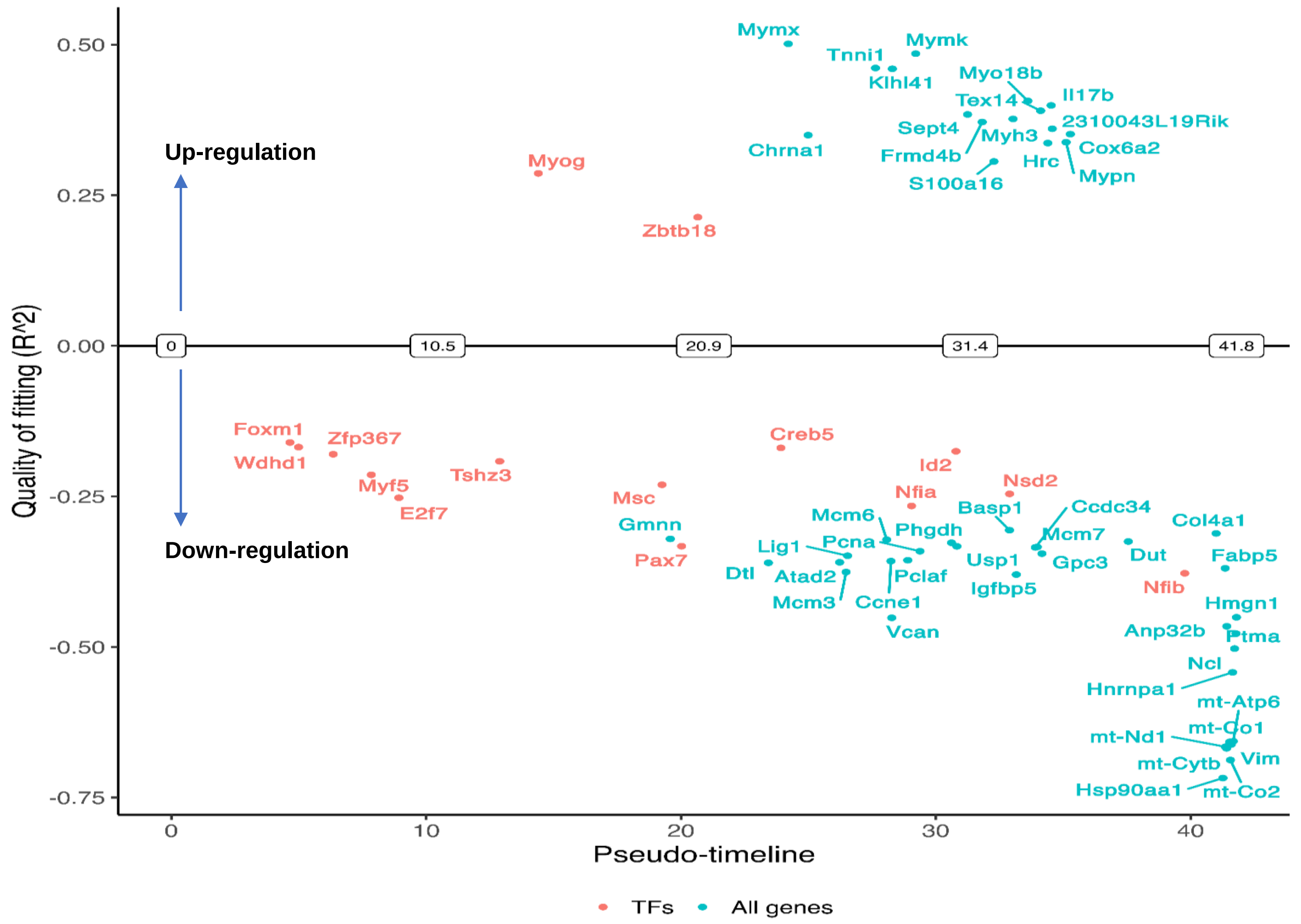

Figure S12. Distinct switching genes of the branch Pre-b to b2 were plotted to pseudo-timeline by McFadden's Pseudo R. The horizontal axis is the pseudo-time, the vertical axis is the Quality of fitting (R^2), the genes turned on with the pseudo-time are above the horizontal axis, and the genes turned off are below the vertical axis.

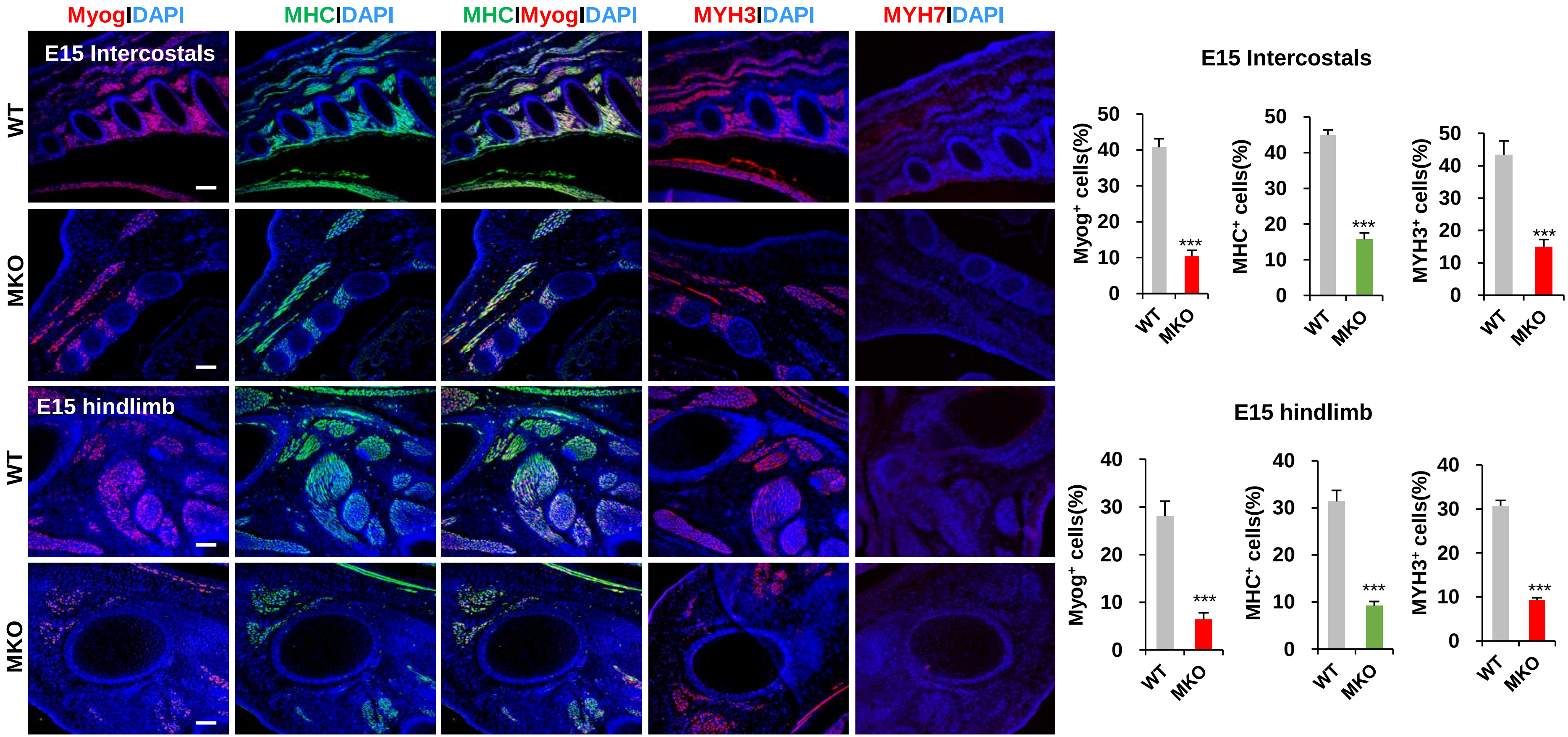

Figure S13. Loss of SRSF2 impaired fetal myoblast differentiation. Representative confocal images of Myog (red), MHC (green), MYH3 (red) and MYH7 (red) immunostaining of intercostal and hindlimb sections of WT and MKO embryos at E15. Scale bars, 100 μm. Qualification was shown on the right bar graphs (n=3). All data are shown as the mean ± SD. 2-tailed Student's t-test.*** $p < 0.001$.

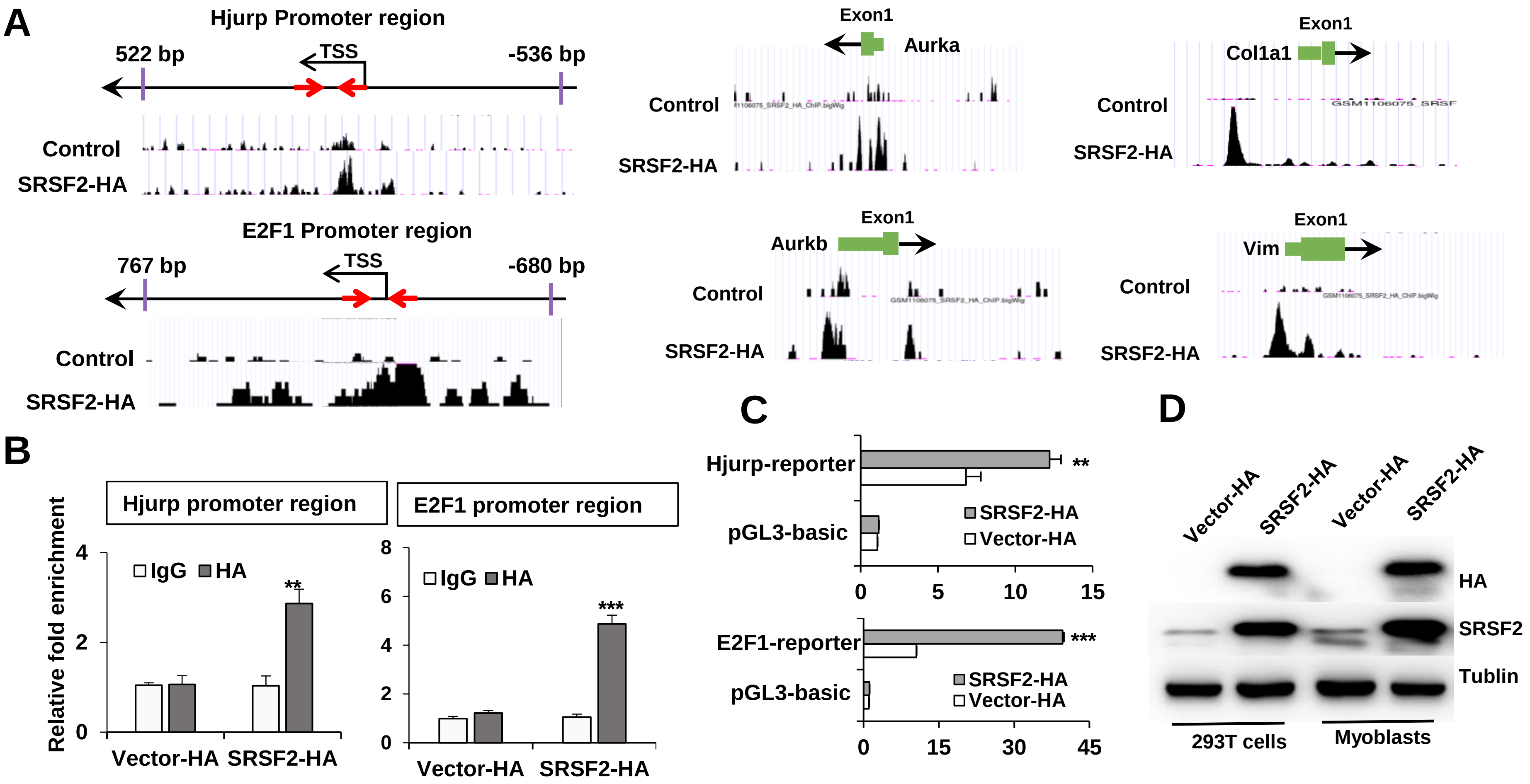

Figure S14. SRSF2 regulated transcription of cell-cycle/cytoskeleton- related genes. A)UCSC genome browser screenshots, illustrating SRSF2 ChIP-seq signals on indicated genes. Note that two pair of primers (red arrows) and their relative positions from the transcription start sites (TSS). B) ChIP-qPCR analysis of SRSF2-bound Hjurp or E2F1 promoter regions in myoblast cells (n=3). Relative enrichment folds were normalized to the IgG group. C) Dual-luciferase (Luci) assay. The luciferase activity expressed by the pGL3-basic control vector was arbitrarily defined as 1 (n=3). D) Representative WB analysis of SRSF2 in myoblasts (B) and 293T cells (C) after 48h transfection (n = 3). All data are presented as mean ± SD. 2-tailed Student's *t*-test, ** indicates P < 0.01,*** indicates P < 0.001.

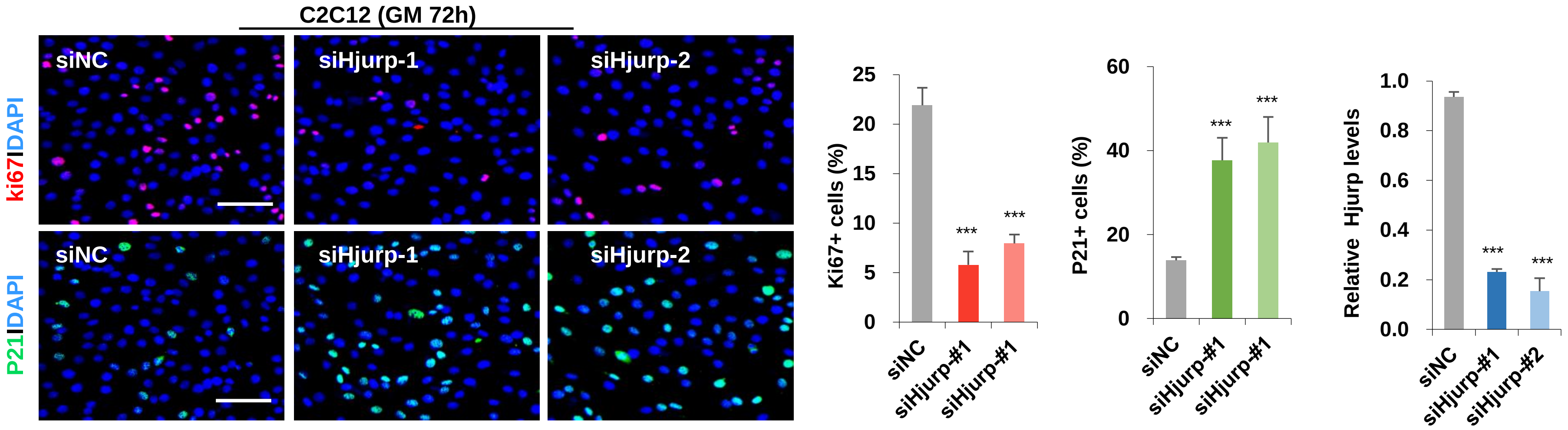

Figure S15. Hjurp knockdown decreased cell proliferation while increased p21 expression. Representative confocal images of p21and Ki67 immunostaining in C2C12 cells (n=3). Scale bars, 100 μm. Hjurp mRNA levels were measured by RT-qPCR (n=3). All data are shown as the mean ± SD. 2-tailed Student's t-test. *p < 0.05.**p < 0.01.***p < 0.001.

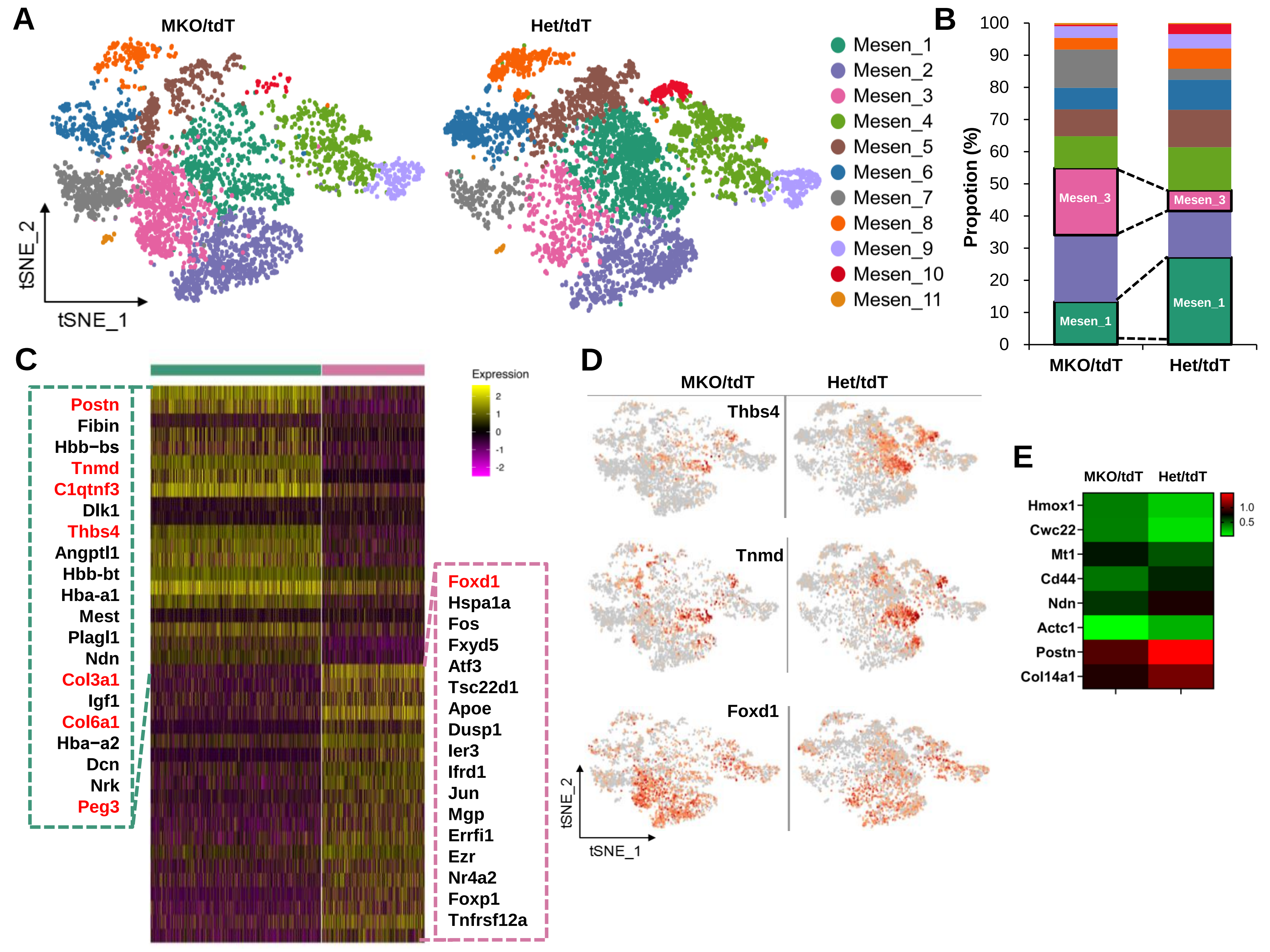
A
MKO/tdT
Het/tdT
tSNE_2
tSNE_1
Mesen_1
Mesen_2
Mesen_3
Mesen_4
Mesen_5
Mesen_6
Mesen_7
Mesen_8
Mesen_9
Mesen_10
Mesen_11
B
Propotion (%)
100
90
80
70
60
50
40
30
20
10
0
Mesen_3
Mesen_1
MKO/tdT
Het/tdT
C
Expression
2
1
0
-1
-2
Postn
Fibin
Hbb−bs
Tnmd
C1qtnf3
Dlk1
Thbs4
Angptl1
Hbb-bt
Hba-a1
Mest
Plagl1
Ndn
Col3a1
Igf1
Col6a1
Hba−a2
Dcn
Nrk
Peg3
Foxd1
Hspa1a
Fos
Fxyd5
Atf3
Tsc22d1
Apoe
Dusp1
Ier3
Ifrd1
Jun
Mgp
Errfi1
Ezr
Nr4a2
Foxp1
Tnfrsf12a
D
MKO/tdT
Het/tdT
Thbs4
Tnmd
Foxd1
tSNE_2
tSNE_1
E
MKO/tdT
Het/tdT
Hmox1
Cwc22
Mt1
Cd44
Ndn
Actc1
Postn
Col14a1
1.0
0.5

Figure S16. Mesen cells subcluster into distinct cell populations. A) t-SNE plot showing projections of sub-clustered mesenchymal cells of MKO/tdT and Het/tdT samples. Each sub-cluster is colored according to cell type. B) Histogram indicating the proportion of sub-clusters in each analyzed sample. Cell subtypes are labeled by colors which correspond to the cell clusters shown in (A). C) A gene expression heatmap showing differentially expressed genes between the Mesen_1 and Mesen_3 subclusters. Yellow corresponds to high expression level; Purple correspond to low expression levels. D) tSNE plot showing the expression of marker genes of Thbs, Tnmd and Foxd1. E) A gene expression heatmap showing differentially expressed genes for subcluster Mesen_1 between MKO/tdT and Het/tdT samples. Red corresponds to high expression level; Green correspond to low expression levels.

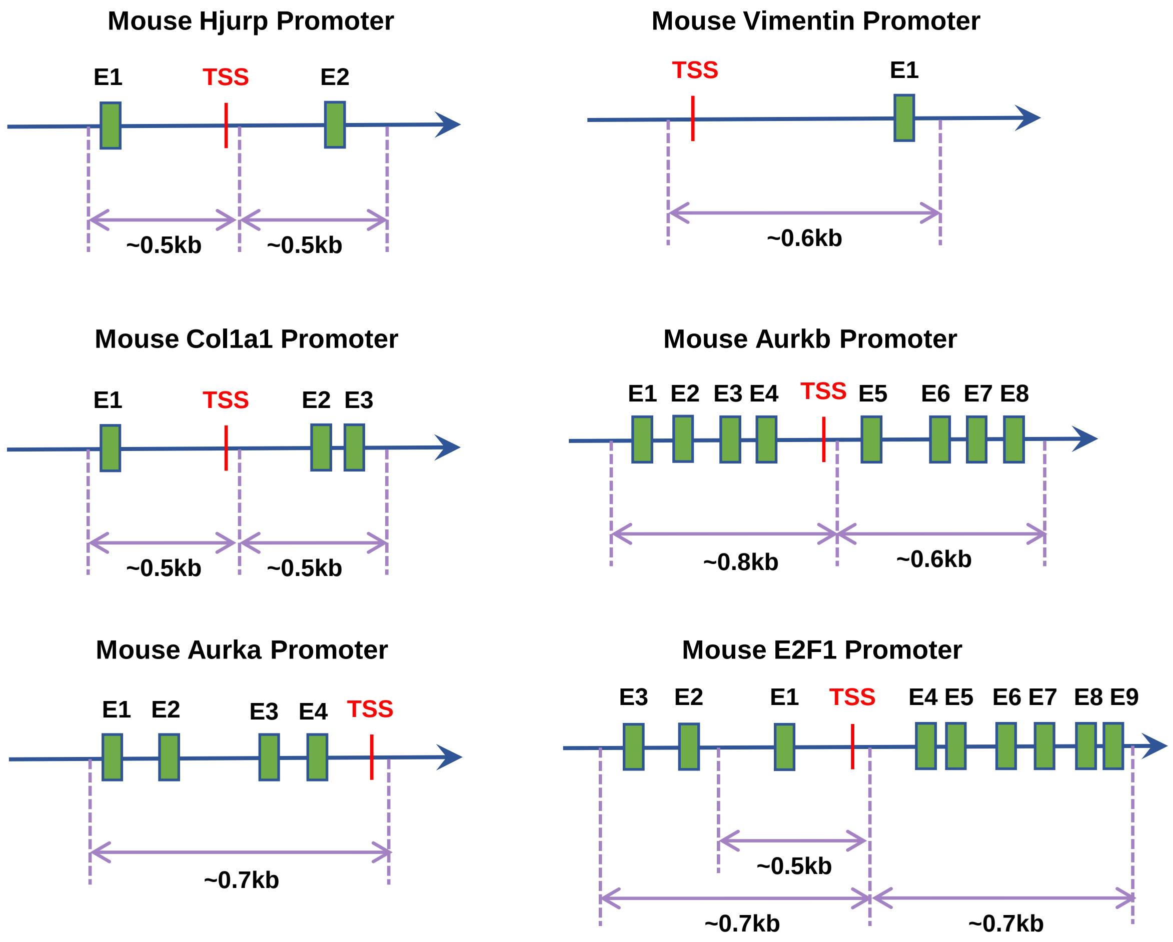

Figure S17. Schematic diagram of potential E-box sequences around the promoter regions of putative SRSF2 targets. Green boxes (E1-E9) stand for potential E-box sites (CANNTG) and their relative positions from the transcription start site of indicated genes (TSS).

Table S1. Antibodies used in WB, IF and ChIP analysis.

Antibody	Origin	Catalog number	Assay
Ki67 Antibody	BD Biosciences	Cat# 550609	IF
p21 Antibody	Abcam	Cat# ab109199	IF
p21 Antibody	Santa Cruz	Cat# sc-817	WB
E2F1 Antibody	Beyotime	Cat# AF6756	WB
cleaved-caspase3 Antibody	Cell Signaling Technology	Cat# 9664	WB
p27 Antibody	Cell Signaling Technology	Cat# 3698	WB
Cyclin B1 Antibody	Cell Signaling Technology	Cat# 4135	WB
PAX7 Antibody	DSHB	Cat# PAX7-c	IF
PAX7 Antibody	Abcam	Cat# ab187339	IF, WB
Myf5 Antibody	Santa Cruz	Cat# sc-302	IF
MyoD Antibody	Santa Cruz	Cat# sc-304	IF
MyoD Antibody	BD Biosciences	Cat# 554130	IF, WB
Myog Antibody	Santa Cruz	Cat# sc-576	IF, WB
Myog Antibody	DSHB	Cat# F5D-c	IF
Myosin heavy chain (MHC) Antibody	Santa Cruz	Cat# sc-20641	IF, WB
Myosin heavy chain (MHC) Antibody	DSHB	Cat# mf-20-s	IF
RFP Antibody	Rockland	Cat# 600-401-379	IF
Retinoblastoma Protein (pRb) Antibody	BD Biosciences	Cat# 554136	WB
Phospho-Rb (Ser807/811) Antibody	Beyotime	Cat# AR092	WB
γH2AX Antibody	Abways	Cat# CY6572	IF, WB
SRSF2 Antibody	Abcam	Cat# ab11826	IF, WB
GAPDH Antibody	Cell Signaling Technology	Cat# 5174	WB
α Tubulin Antibody	Santa Cruz	Cat# sc-32293	WB
β-Actin Antibody	Santa Cruz	Cat# sc47778	WB
Anti-HA tag	Abcam	Cat# ab9110	WB, ChIP
Myosin heavy chain Type I (MYH7) Antibody	DSHB	Cat# BA-D5	IF
myosin heavy chain3 (MYH3) Antibody	Santa Cruz	Cat# sc-324154	IF
Goat anti-Mouse IgG (H+L) Cross-Adsorbed Secondary antibody, Alexa Fluor 488	Thermo Fisher Scientific	Cat# A-11001	IF

Goat anti-Mouse IgG (H+L) Cross-Adsorbed Secondary antibody, Alexa Fluor 546	Thermo Fisher Scientific	Cat# A-11003	IF
Goat anti-Rabbit IgG (H+L) Cross-Adsorbed Secondary antibody, Alexa Fluor 488	Thermo Fisher Scientific	Cat# A-11008	IF
Goat anti-Rabbit IgG (H+L) Cross-Adsorbed Secondary antibody, Alexa Fluor 546	Thermo Fisher Scientific	Cat# A-11010	IF
Donkey anti-Goat IgG (H+L) Cross-Adsorbed Secondary Antibody, Alexa Fluor 647	Thermo Fisher Scientific	Cat# A-21447	IF

Table S2. Primer sequences used for mRNA expression analysis

Gene	Forward (5'–3')	Reverse (5'–3')	Size (bp)
SRSF2	TCCTCCTCCGTCTCCAGATC	GCGACCTAGACTTGGACTCTC	97
Hjurp	TCATGACTTGGGTTCCTTCC	TAGCCACAATGGCCATATCA	162
MyoD	CGCTCCAACTGCTCTGATG	CTGTAGTAGGCGGTGTCGTA	89
Myf5	TGCCATCCGCTACATTGAGA	CGTCAGAGCAGTTGGAGGT	119
Pax7	AATCAGCTTGGTGGGGTCTT	ATCGGCACAGAATCTTGGAG	154
Rplp0	TAAAGACTGGAGACAAGGTGGGAG	AGAAAGCGAGAGTGCAGGGC	166

Table S3. Primer sequences used for mice genotyping

Gene	Forward (5'–3')	Reverse (5'–3')	Size (bp)
SRSF2-GT	GGTTATTTGGCCAAGAATCAC	GGTTATTTGGCCAAGAATCAC	300, 400
Myf5-cre-GT	CGTAGACGCCTGAAGAAGGTCAACCA	CACATTAGAAAACCTGCCAACACC	400
		ACGAAGTTATTAGGTCCCTCGAC	600
tdT-GT	CTCTGCTGCCTCCTGGCTTCT	CGAGGCGGATCACAAGCAATA	300
		TCAATGGGCGGGGGTCGTT	250

Table S4. Primer sequences used for ChIP-qPCR

Gene	Forward (5'–3')	Reverse (5'–3')	Size (bp)
Hjurp-ChIP	GCCACGTTTGATCTGTGGAC	AAGCGGCTACTGCTTTCCTT	128
E2F1-ChIP	GTAAAAGTGGCCCGGACTTTG	GATGACGATCTGCGAGGAGT	232

Table S5. Primer sequences used for pGL3-based reporter plasmid construction in dual-luciferase activity assay

Gene	Forward (5’–3’)	Reverse (5’–3’)	Size (bp)
E2F1-promoter	CCGCTCGAGGGATGACCTGAACTGGGAAG	CCCAAGCTTACTCCCTAGCCATAGGTTCT	1483
Hjurp-promoter	CCGCTCGAGTTCCATTAATTCCCATATCG	CCCAAGCTTTTCAAGGTCCCTAGGAGCAA	1058

Table S6. siRNA sequences used for RNA interference.

	Sense	Antisense
SRSF2 siRNA1	CGAAGAUCCAAGUCCAAGUTT	ACUUGGACUUGGAUCUUCGTT
SRSF2 siRNA2	UCCAGAUCAACCUCCAAGUTT	ACUUGGAGGUUGAUCUGGATT
p21 siRNA	GAGCAGUUGCGCCGUGAUUTT	AAUCACGGCGCAACUGCUCTT
siHjurp siRNA1	GCUGAUAUGGCCAUUGUGGTT	CCACAAUGGCCAUAUCAGCTT
siHjurp siRNA2	GCAUCAGCAGCUUAAGGAATT	UUCCUUAAGCUGCUGAUGCTT
Negative control	UUCUCCGAACGUGUCACGUTT	ACGUGACACGUUCGGAGAATT
